# Supplementary material for: Establishing superfine nanofibrils for robust polyelectrolyte artificial spider silk and powerful artificial muscles
Source: Nat Commun. 2024 Apr 25;15:3485. doi: 10.1038/s41467-024-47796-2 (PMC11045855; doi:10.1038/s41467-024-47796-2)
Supplement: Supplementary file 1 — Supplementary Information [file 41467_2024_47796_MOESM1_ESM.pdf]

Supplementary Information for

Establishing Superfine Nanofibrils for Robust Polyelectrolyte Artificial Spider Silk and Powerful Artificial Muscles

Wenqian He[1], Meilin Wang[1], Guangkai Mei[1], Shiyong Liu[1], Abdul Qadeer Khan[1], Chao Li[1], Danyang Feng[1], Zihao Su[1], Lili Bao[2], Ge Wang[1], Enzhao Liu[3], Yutian Zhu[4], Jie Bai[5], Meifang Zhu[6]*, Xiang Zhou[2]*, Zunfeng Liu[1]*

[1]State Key Laboratory of Medicinal Chemical Biology, Key Laboratory of Functional Polymer Materials, College of Chemistry, Nankai University, Tianjin 300071, China

[2]Department of Science, China Pharmaceutical University, Nanjing 211198, China

[3]Tianjin Key Laboratory of Ionic-Molecular Function of Cardiovascular disease, Department of Cardiology, Tianjin Institute of Cardiology, the Second Hospital of Tianjin Medical University, Tianjin, 300211, China

[4]College of Materials, Chemistry and Chemical Engineering, Hangzhou Normal University, Hangzhou, 311121, China

[5]Inner Mongolia Univ Technol, Chem Engn Coll, Hohhot 010051, China

[6]State Key Laboratory for Modification of Chemical Fibers and Polymer Materials, College of Materials Science and Engineering, Donghua University, Shanghai, 201620 China

Corresponding authors' emails: liuzunfeng@nankai.edu.cn (Z. Liu); zhouxiang@cpu.edu.cn (X. Zhou); zhumf@dhu.edu.cn (M. Zhu)

This file contains the following contents:

Supplementary Methods 1 (Page 2–8)

Supplementary Figures 1 to 35 (Pages 9–27)

Supplementary Tables 3 to 11 (Page 28–34)

Supplementary Data 1 (Page 34)

References (Page 35–38)

1. Supplementary Methods

Characterization. The SEM images were obtained using a scanning electron microscope (MERLIN, Zeiss). The AFM images were obtained using an atomic force microscope (modeled Dimension Icon) in tapping mode; the tip radius was 10 nm (1 Ohm Silicon). The POM images were obtained using a polarizing microscope (Shunyu CX40P). The optical images were obtained using a metallographic microscope in reflective mode (Chenxing CXML1000). The XRD patterns were obtained using an X-Ray diffractometer (Rigaku Mini Flex) in the angular range from 3° to 50° at a scan rate of 3° min^{-1}. The FTIR spectra were obtained using a Nicolet iS50 FTIR spectrometer with an attenuated total reflectance mode in the wavelength range of 400–4000 cm^{-1}. Thermogravimetric analysis (TGA) was performed using a thermogravimeter (Netzsch Mettler-Toledo 209) in N_2 atmosphere at a flow rate of 10°C min^{-1}. The stress relaxation measurements were performed using a mechanical tester (CTM-2GD) with a pre-strain of 5% in the temperature range of 20°C–50°C at an environmental RH of 35%.

WAXS measurements were performed using a Xeuss 2.0 system (Xenocs SA, France) equipped with a 2D detector (600×600 pixels with a pixel size of 172×172 μm^2) employing Cu Kα radiation with a wavelength of 1.54 Å. The sample-to-detector distance was 112 mm, and the data acquisition time was 600 s. The orientation degree of the polymer chains in the fiber length direction was evaluated using f_m. The details of the calculations were as follows: Firstly, the azimuthal curve was obtained by integrating the diffraction intensity of the 2D WAXS pattern over the azimuthal angle (Φ) using the general area detector diffraction system (GADDS) software for the 2θ angles. Secondly, the full width at half maximum of the peak, θ_h, was obtained, and f_m was calculated as $f_m = (180° - \theta_h)/180°$.

For investigating the alignment degree of the nanofibrils of 80-μm-diameter PAF with different α and of 30-μm-diameter PAF with different twist densities, SAXS measurements were performed at the BL16B1 beamline of the Shanghai Synchronization Radiation Facility. The wavelength was λ = 1.24 Å, and a charge-

coupled device (CCD) X-ray detector (1475×1679 pixels with a pixel size of 172×172 μm^2) was employed to collect the data. The sample-to-detector distance was 2330 mm, the data acquisition time was 5 s, and the 2D scattering images were analyzed using the Fit2D software provided by the European Synchronization Radiation Facility. For investigating the alignment degree of the nanofibrils of 95-μm-diameter PAF with different twist densities, SAXS measurements were performed using a Xeuss 2.0 system employing Cu Kα radiation with a wavelength of 1.54 Å. The sample-to-detector distance was 2274 mm, and the data acquisition time was 600 s.

The orientation degree of the nanofibrils (f_n) was calculated from the azimuthal-integrated intensity distribution curves of the 2D SAXS patterns using the following equations:

$$f_n=\frac{3(\cos^2\Phi)-1}{2} \quad (1)$$

and

$$\cos^2\Phi=\frac{\int_0^{\Pi/2} I(\Phi)\,\cos^2\Phi\,\sin\Phi\,d\Phi}{\int_0^{\Pi/2} I(\Phi)\,\sin\Phi\,d\Phi}\;\Phi \quad (2)$$

where Φ is the azimuthal angle, and $I(\Phi)$ is the one-dimensional intensity distribution along Φ. $\langle\cos^2\Phi\rangle$ is calculated by integrating the intensity of the specific 2θ diffraction peak along Φ.

All the tensile tests were carried out on a single PAF on an Instron mechanical tester. The PAF for mechanical test were taped onto the paper frames with a gauge length of 6 mm. The environmental temperature was 25°C, and the relative humidity was 10%. The final mechanical properties were the average values of 5 independent tests.

The water content of the PAF_α was obtained by calculating the mass difference between the initial PAF_α (m_0, dried at 60°C for 3 h) and the PAF_α vacuum dried at 100°C for 12 h (m_{dry}) divided by the initial mass m_0.

$$C=\frac{m_0-m_{dry}}{m_0}\times100\% \quad (3)$$

The work capacity (J g^{-1}) can then be calculated directly:

$$W = \frac{m_{load}}{m_0}\mathbf{g}l \quad (4)$$

where m_{load} is the loading mass, m_0 is the fiber mass, l is the displacement of the loading mass during actuation, and **g** is the acceleration constant of gravity.

To make a notch on the fiber, a sharp blade was employed to make a 15-μm-deep single-edge cut in the radial direction of a 100-μm-diameter PAF_α that has been dried for 3 h in 60°C. Then, the fiber with notch was then subjected to mechanical test at a stretch rate of 5 mm min^{-1}.

The actuation properties were characterized by two methods in the revised manuscript, as follows. In the method 1, the actuation of the PAF_α was characterized by lifting a load by heating the fiber to an elevated temperature. The PAF_α with radius of r and length l_0 was loaded with a mass of m_{load}, and then the PAF_α was heated to an elevated temperature. The PAF_α contracted to length of l_1. The actuation stress was calculated as $m_{load}\mathbf{g}/\pi r^2$, and the actuation strain was calculated as $(l_0 - l_1)/l_0 \times 100\%$.

In the method 2 (added in the revised manuscript), the actuation of the PAF_α was characterized by directly measuring the contraction force on the mechanical tester by the heating the fiber to an elevated temperature. The PAF_α was both-end tethered on the clamps of the mechanical tester. Then, the PAF_α was heated to an elevated temperature to cause contractive force (F), which is measured by the mechanical tester. The actuation stress was calculated as $F/\pi r^2$.

For twist insertion, the wet as-obtained PAF_α before drying was employed. One end of a 25-mm-long PAF_α was vertically connected to an 80-step servomotor, and the other end was isobarically loaded (constant load 3.53 MPa) with a stainless-steel ring. An iron rod was inserted into the stainless-steel ring to prevent the PAF_α from rotating during twist insertion. Then, twist was inserted by rotation of the servomotor. Different twist densities were inserted in the fibers. After twist insertion, the PAF_α was dried for 3 h in 60°C for mechanical properties tests.

Calculation of the dissociation degree (α)

The pH values of the acrylic acid solution were obtained using an Orion 3-Star Benchtop pH Meter. The dissociation degree α can be calculated as follows:

$$\alpha = \frac{[CH_2=CH_2COO^-]}{[CH_2=CH_2COO^-]+[CH_2=CH_2COOH]}, \quad (5)$$

where $[CH2 = CH2COO-]$ and $[CH2 = CH2COOH]$ are the molar concentrations of $CH_2{=}CH_2COO^-$ and $CH_2{=}CH_2COOH$, respectively.

$$K_a=\frac{[CH_2=CH_2COO^-][H^+]}{[CH_2=CH_2COOH]}, \quad (6)$$

where K_a is the dissociation constant, and $[H^+]$ is the molar concentration of protons.

$$\alpha=\frac{K_a}{K_a+[H^+]}, \quad (7)$$

$$\alpha=\frac{K_a/[H^+]}{K_a/[H^+]+1}, \quad (8)$$

$$[H^+]=10^{-pH}, \quad (9)$$

$$K_a=10^{-pK_a}, \quad (10)$$

$$\alpha=\frac{10^{-pK_a}/10^{-pH}}{10^{-pK_a}/10^{-pH}+1}=\frac{10^{(pH-pK_a)}}{10^{(pH-pK_a)}+1}, \quad (11)$$

where the pK_a value of the $CH_2{=}CH_2COOH$ solution is 4.752.

Calculation of the free energy of polymer chain relaxation (E_r) of the PAF_α

The free energy of polymer chain relaxation (E_r) of the PAF_α was obtained by stretching the fiber to a pre-strain of 5% using a mechanical tester, and then the time it took for the stress to decrease to $1/e$ of the initial stress was measured at different environmental temperatures (Supplementary Fig. 20). E_r was obtained from the *Arrhenius* equation, that is:

$$\ln \tau = E_a/RT + \ln\tau_0, \tag{12}$$

where R = 8.314 J mol^{-1} K^{-1} is the gas constant.

Coarse-grained MD simulations

Coarse-grained model

The bead division and mapping of the coarse-grained model of the PAF_α were inspired by the Martini 3 force field.[1] The Martini 3 force field, which is a new version of the general coarse-grained force field, provides more parameters for coarse-grained beads to take into account different chemical properties, and beads of different sizes are employed to map various chemical groups with different masses. Therefore, an accurate and flexible mapping of a complex chemical structure can be performed. The four carbon atoms on the main chain were mapped to a regular bead; the carboxyl and carboxylate groups of the side chains and metal cations were mapped to the corresponding small beads (Supplementary Table 1).[2] The two repeating units of the PAF_α were mapped to one repeating unit of a partially dissociated coarse-grained polymer chain composed of four beads. Seventy-five polymer chains were placed in a box, with 250 repeating units for each molecular chain. The molecular chain of the PAF_α was generated using a random walking model. Systems with dissociation degree (α) values of 0%, 5%, 25%, and 50% were established, and the number of free cations was consistent with the number of carboxylate anions to ensure the charge neutrality of each system. The random distribution of COOH and COO^- was employed to simulate the side chain structure after neutralization.

MD Force Field Parameters

The truncated, and shifted Lennard–Jones (LJ) potential for the non-bonded interactions between all beads is expressed as follows:

$$U_{LJ}(r_{ij}) = 4\epsilon_{ij}\left[\frac{\sigma_{ij}^{12}}{r_{ij}} - \frac{\sigma_{ij}^{6}}{r_{ij}}\right] - U_{LJ}(r_{cutoff}) \tag{13}$$

where $r_{ij} < r_{cutoff}$, $U_{LJ}(r_{cutoff})$ is a constant that ensures that the non-bonding potential energy is continuous at the cutoff distance. ϵ_{ij} is the energy parameter, σ_{ij} represents the

length parameter, and r_{ij} is the distance between the pairs of the coarse-gained model. The energy and distance parameters (Supplementary Table 2) were derived from the force field data provided by the Martini 3 force field.

For pairs of beads with identical charges, the distance cutoff was set to $2^{1/6}\sigma_{ij}$, while for the other bead pairs, the cutoff was set to 2.5 σ_{ij}. The LJ potential energy function was shifted up at the cutoff, so that the potential energy at this point was zero. The finite extensible nonlinear elastic (FENE) potential was employed to describe the bead spring polymer model:

$$U_{FENE}=0.5KR_0^2 \ln\left[1-\left(\frac{r}{R_0}\right)^2\right] \quad (14)$$

where U_{FENE} is the potential term related to the covalent bonds, and r is the distance between the connected beads. The maximum length of the bond R_0 was set to 1.5σ to avoid chain crossing. The spring constant was $K = 30\varepsilon/\sigma_{ij}{}^2$. The beads of the carboxylate anions and metal cations experience a long-range Columbic interaction, that is:

$$U_{Coulomb}\left(r_{ij}\right)=\frac{Cq_iq_j}{\varepsilon_r r_{ij}} \quad (15)$$

where $r < r_c$, U_{Coulomb} is the potential term related to the covalent bonds, C is an energy-conversion constant, q_i and q_j are the charges on the beads i and j, respectively, and ε_r is the dielectric constant, which was set to 0.028 according to Hall's work.[3]

MD Simulations

The MD simulations were carried out using the LAMMPS software package.[4-5] A Nosé–Hoover thermostat and barostat were employed to execute simulations in the NPT (constant pressure and constant temperature) ensemble, which was used to obtain a reasonable density as well as the box size to simulate the system in the canonical ensemble (with the same thermostat and no barostat). Specifically, a temperature damping parameter of 0.5τ (reduced time unit) was employed to maintain the temperature at $1.25T^*$, where the reduced temperature $T^* = 1\ kT/\varepsilon$, k is the Boltzmann constant, T is the true temperature, and ε is an energy parameter. A pressure damping parameter of 5τ was employed to maintain the pressure at zero. Periodic boundary

conditions and the velocity Verlet algorithm with a time step of 0.005τ were used in all the simulations.

The simulation details were as follows. Firstly, the soft potential was used to run the simulation for 1000τ to eliminate the particle overlap of the initial model under the NVT ensemble. Then, the soft potential was replaced by a mixed LJ and Coulombic potential, and each system was equilibrated for $5\times10^4\tau$ under the NPT ensemble to allow the density to reach a stable state. Subsequently, the barostat was removed, and the system was equilibrated for $2\times10^5\tau$ under the NVT ensemble. After a long relaxation time, the MSD of the molecular chains became greater than $3R_g^2$, indicating that the structure of the aggregation state had become relatively stable. Finally, the MSD (Supplementary Fig. 18b) calculation and cluster analysis were performed on the basis of the stable structure.

The cluster was defined as a group of closely aggregated particles. Any two particles from the same cluster could be connected through a continuous path. By contrast, if there was no continuous path from one particle to another on an adjacent network, the two particles were considered not to belong to the same cluster. Here, if the distance between two particles was less than or equal to σ_{ij}, it was considered that the two particles were connected by continuous paths and belonged to the same cluster. If the number of particles in this aggregate was greater than N (N = 10 in this work), it was considered that the aggregation formed a cluster. The trajectories and cluster analysis were visualized using the OVITO software (Supplementary Fig. 19).

2. Supplementary Figures

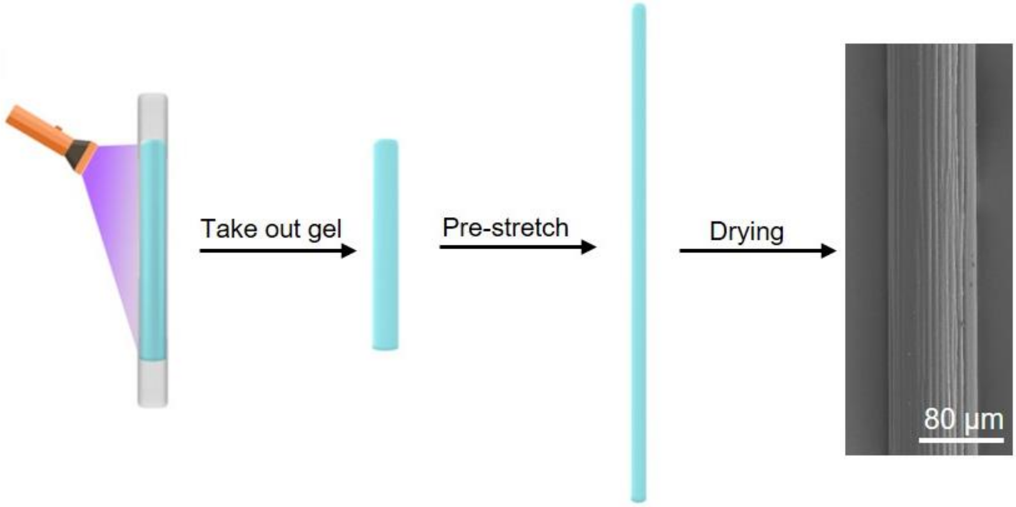

Supplementary Figure 1. The preparation of PAF_{α} artificial spider silk in a capillary tube.

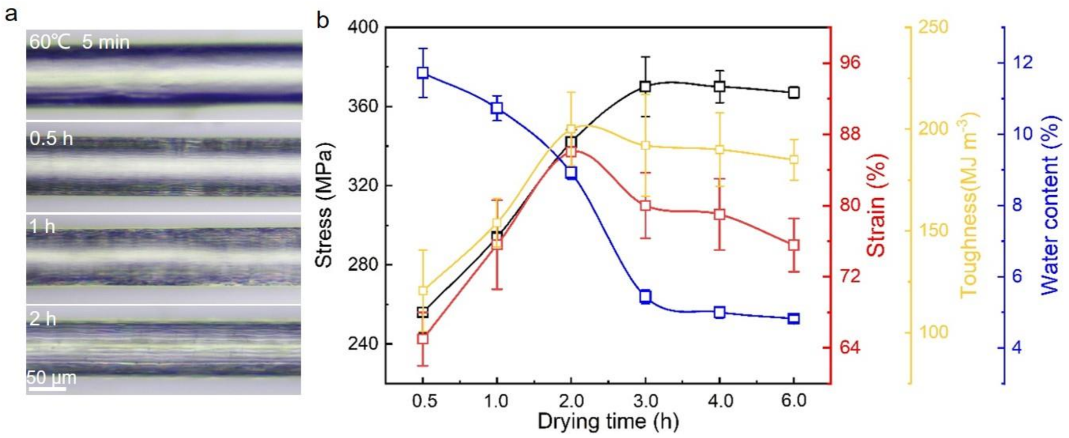

Supplementary Figure 2. Metallographic microscopy images and mechanical properties of PAF for different drying time. (a) The sheath–core structure of PAF for different drying time. As the drying time increased from 5 min to 1h, the fiber sheath ratio increased from 31% to 77%, and the fiber almost completely dried at 2 h. (**b**) Breaking stress, breaking strain, toughness, and water content of $PAF_{0.17\%}$ dried at 60°C for different time. The error bars for (b) represent mean ± SD (*n*=5 independent samples).

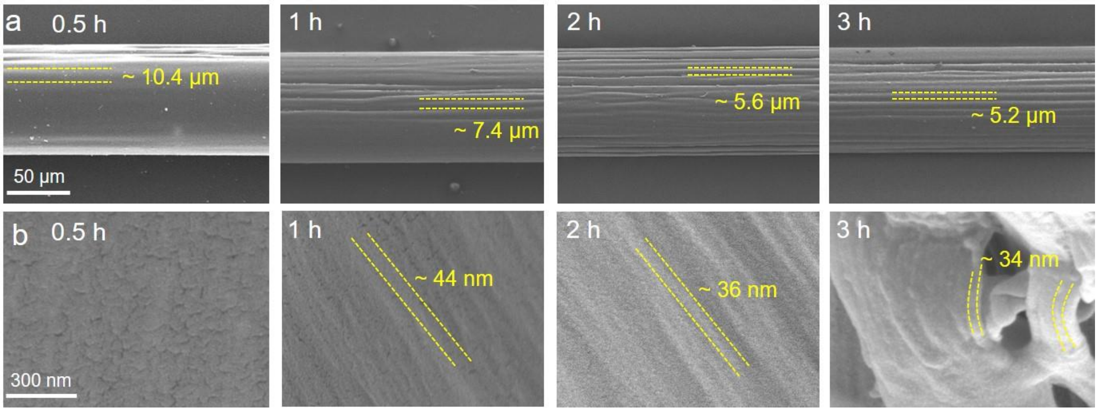

Supplementary Figure 3. SEM images of PAF surface and longitudinal cross section. (**a**) Surface microstrips and (**b**) longitudinal sectional nanofibrils of the $PAF_{0.17\%}$ dried at 60°C for different time.

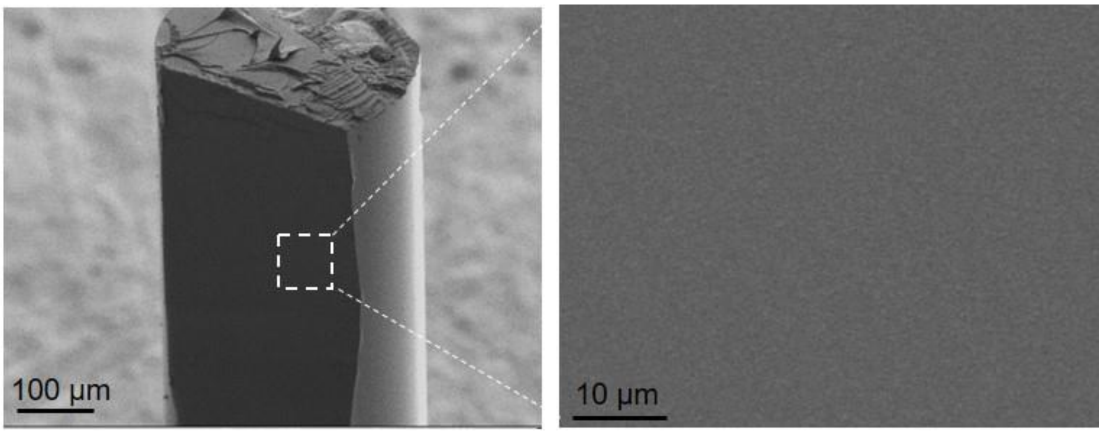

Supplementary Figure 4. (a) Low and (b) high magnification longitudinal sectional SEM images of the $PAF_{0.17\%}$ dried at 60°C without a pre-strain, exhibiting negligible nanofibrils observed in the longitudinal section.

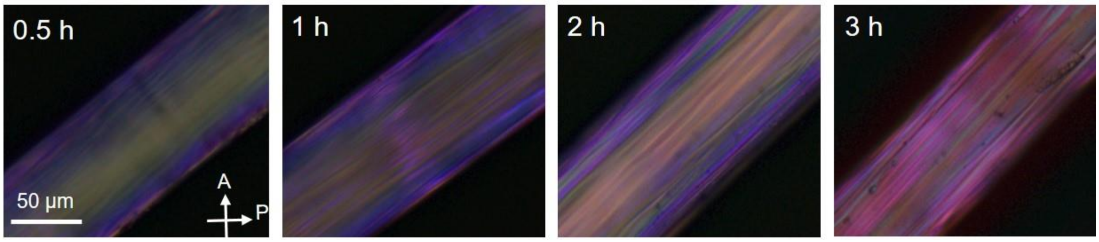

Supplementary Figure 5. POM images of the $PAF_{0.17\%}$ dried at 60°C for different time.

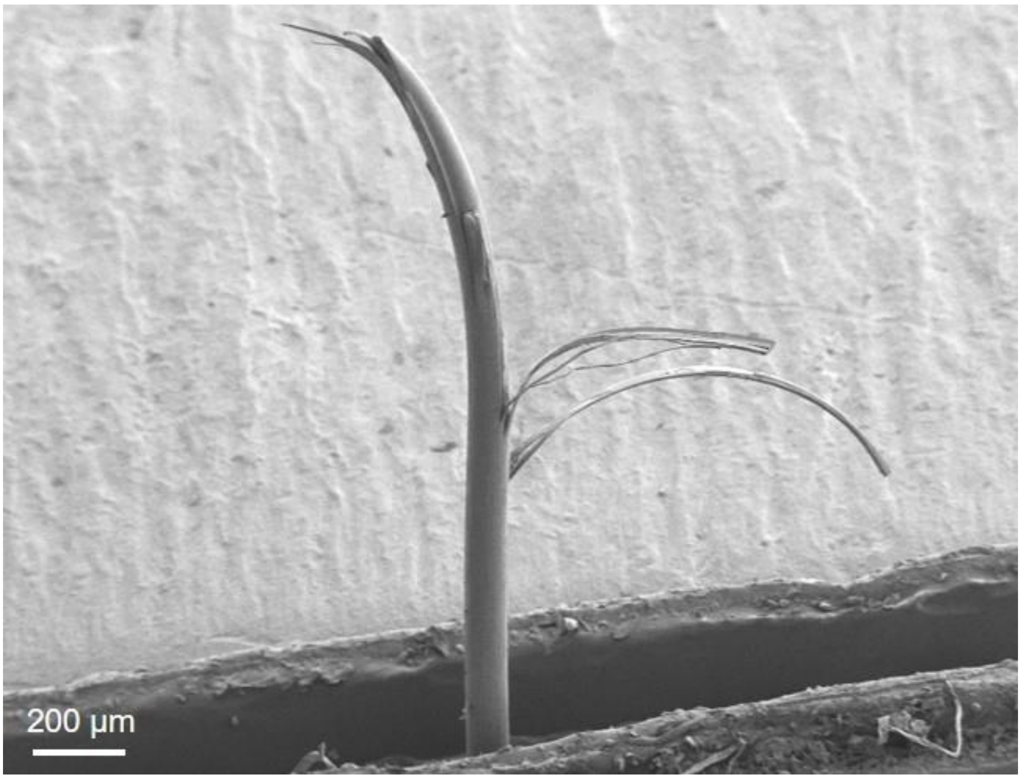

Supplementary Figure 6. The SEM image of a fractured PAF$_{0.17\%}$ showing nanofibrils pulling out by mechanical stretch.

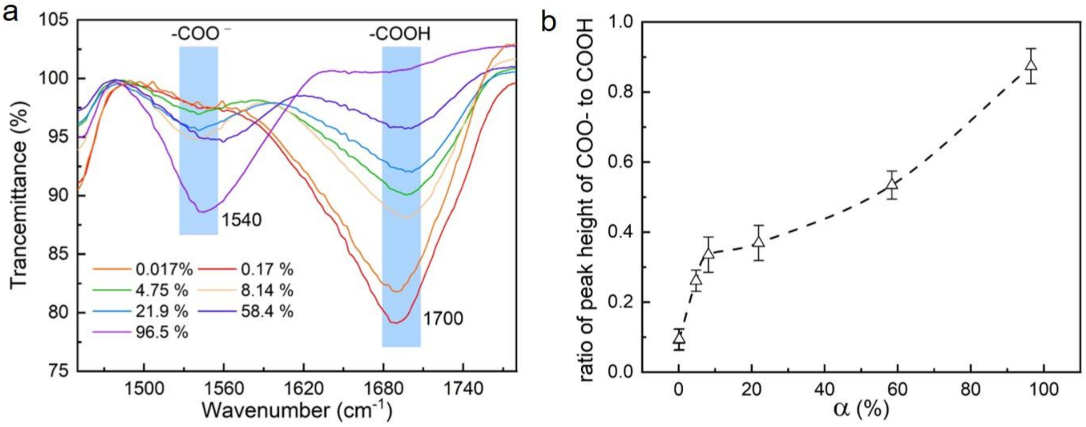

Supplementary Figure 7. (a) FTIR of PAF$_\alpha$ for different α values. (b) The ratio of peak height of the –COO$^-$ (1540 cm^{-1}) to that of the –COOH (1700 cm^{-1}) as a function of α values. The error bars for (b) represent mean ± SD (n=3 independent samples).

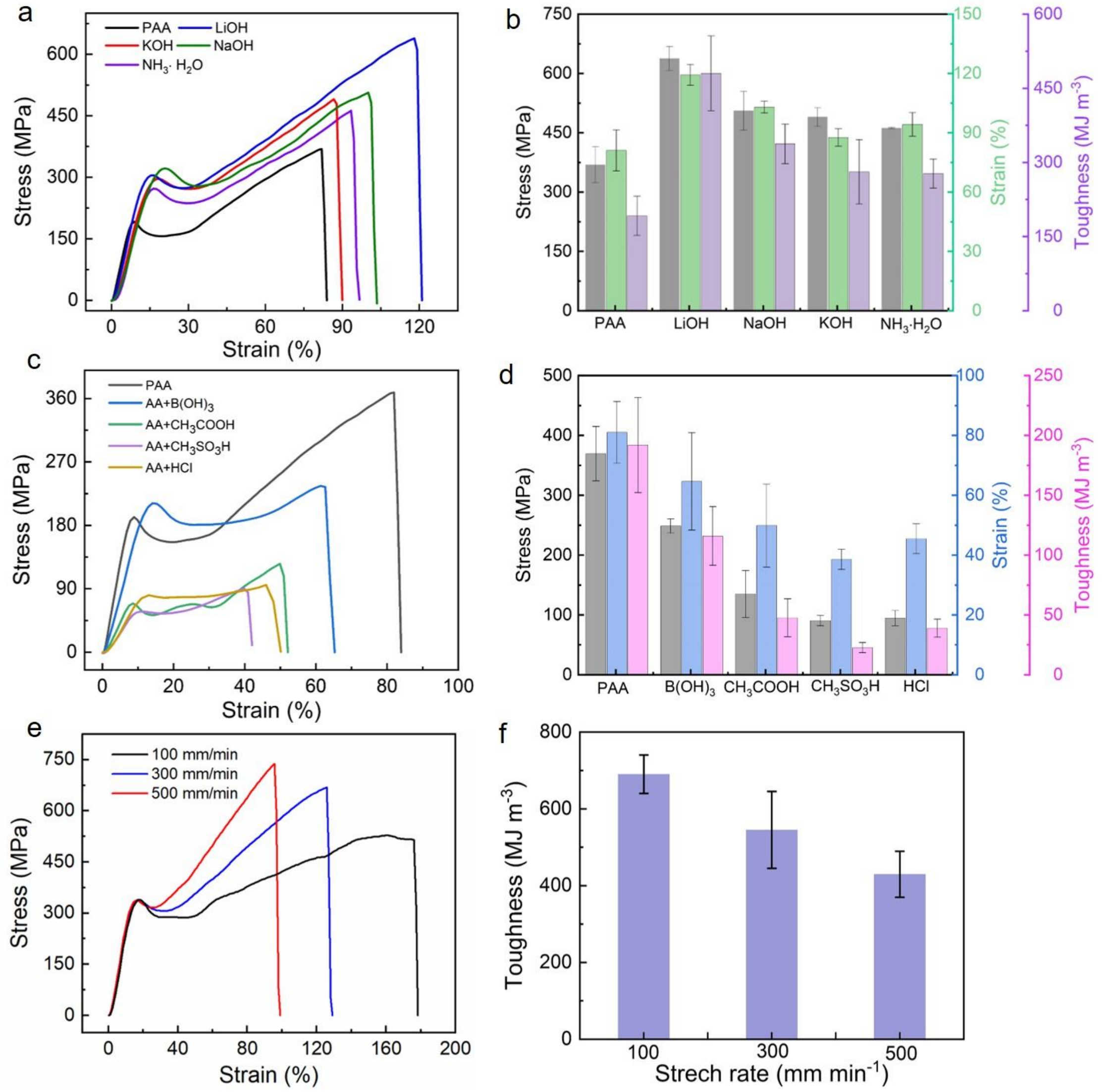

Supplementary Figure 8. The mechanical properties of the PAF by adding different types of bases and acids. (**a**) Stress–strain curves and (**b**) breaking stress, fracture strain, and toughness of the PAF by adding different types of bases; (**c**) Stress–strain curves and (**d**) breaking stress, fracture strain, and toughness of the PAF by adding different types of acids; (**e**) Stress–strain curves and (**f**) toughness of the $PAF_{4.75\%}$ at different stretch rates. If not specified, in this and the following figures, the fiber diameter was 80 μm. The stretch rate in (a) to (d) was 200 mm min^{-1}. The error bars for (b), (d) and (f) represent mean ± SD (n=5 independent samples).

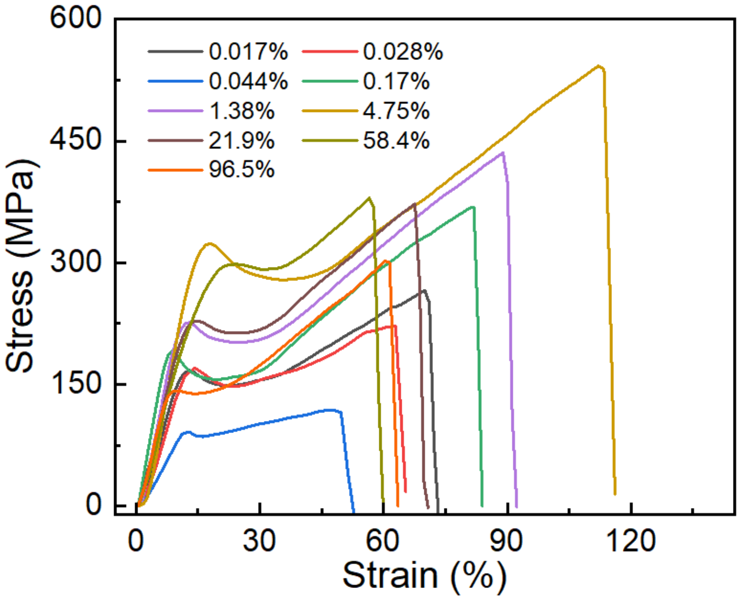

Supplementary Figure 9. Stress–strain curves of the PAF$_\alpha$ for different α values. The fiber diameter was 80 μm. The stretch rate in the mechanical tests was 200 mm min^{-1}.

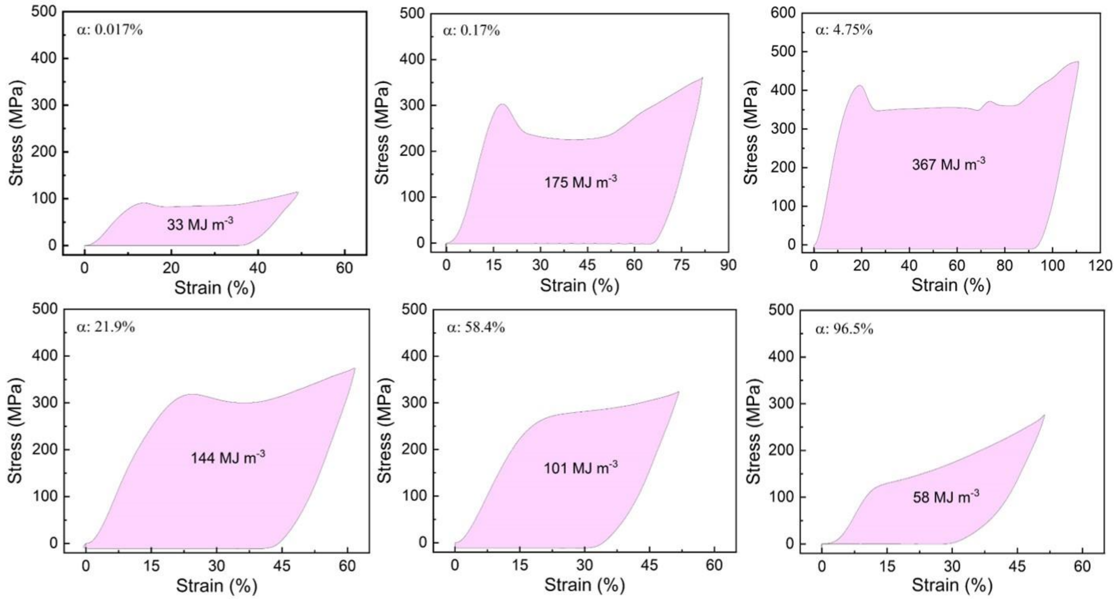

Supplementary Figure 10. Stress–strain curves of the PAF$_\alpha$ for different α values, showing the dissipation energy for loading and unloading.

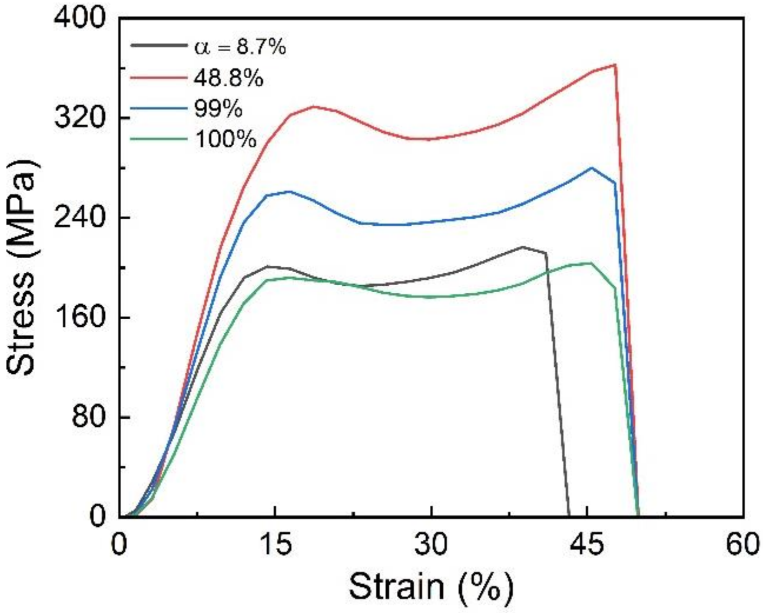

Supplementary Figure 11. tress–strain curve of the p(AM-*co*-DMAEMA) fibers with different α by adding hydrochloric acids. The fiber diameter was 55 μm, the stretch rate during mechanical test was 200 mm min^{-1}.

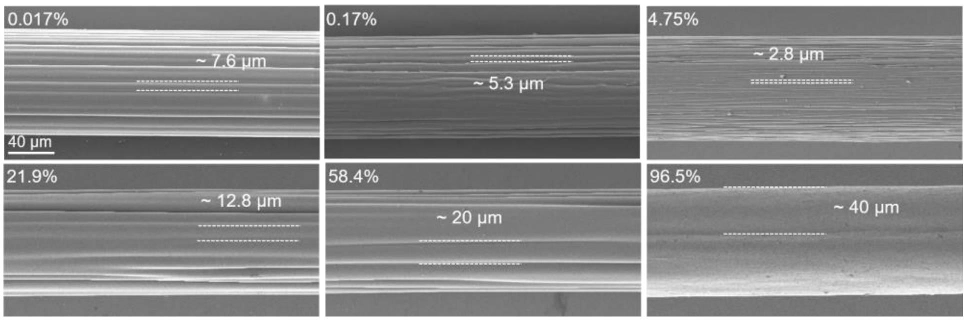

Supplementary Figure 12. Surface view of SEM images of the PAF$_\alpha$ with different α values dried at 60°C for 3 h.

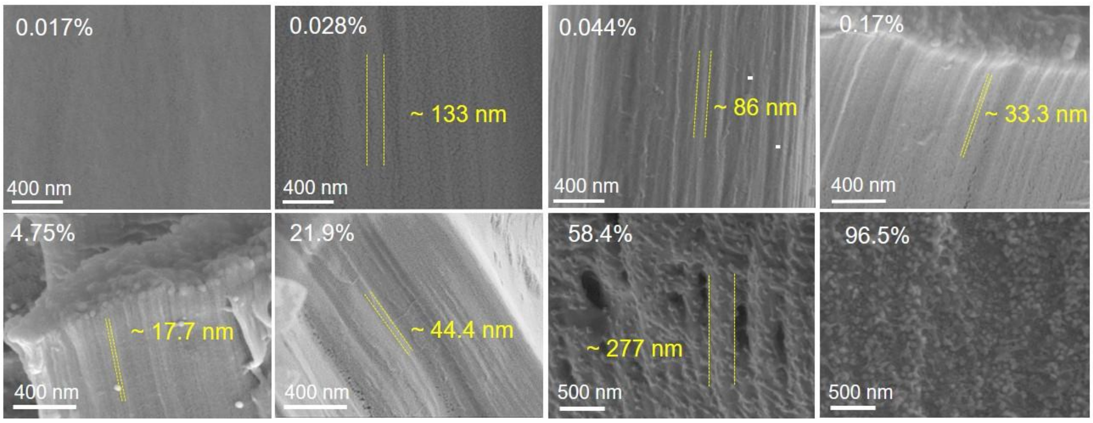

Supplementary Figure 13. Longitudinal sectional view of SEM images of the PAF$_\alpha$ with different α values dried at 60°C for 3 h.

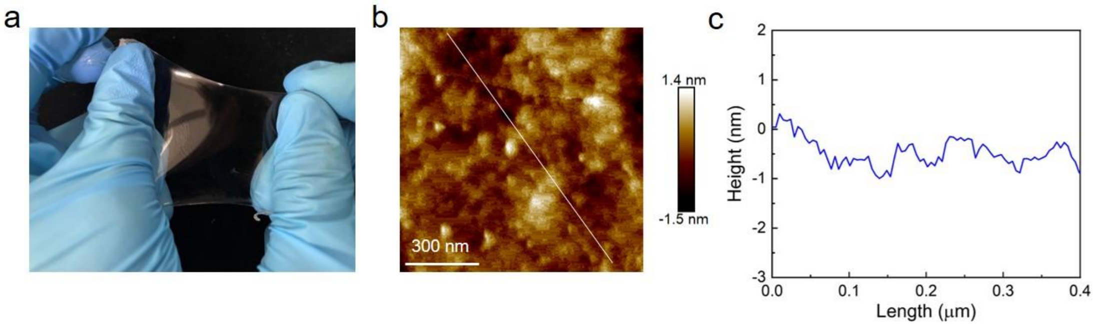

Supplementary Figure 14. The preparation and characterization of the polyacrylic acid film. (**a**) Optical photograph, (**b**) AFM height image, and (**c**) height profile along the with line in (b) of polyacrylic acid film (20 mm × 20 mm × 12 µm) that was prepared by bi-axial stretching of a polyacrylic gel (10 mm × 10 mm × 0.2 mm).

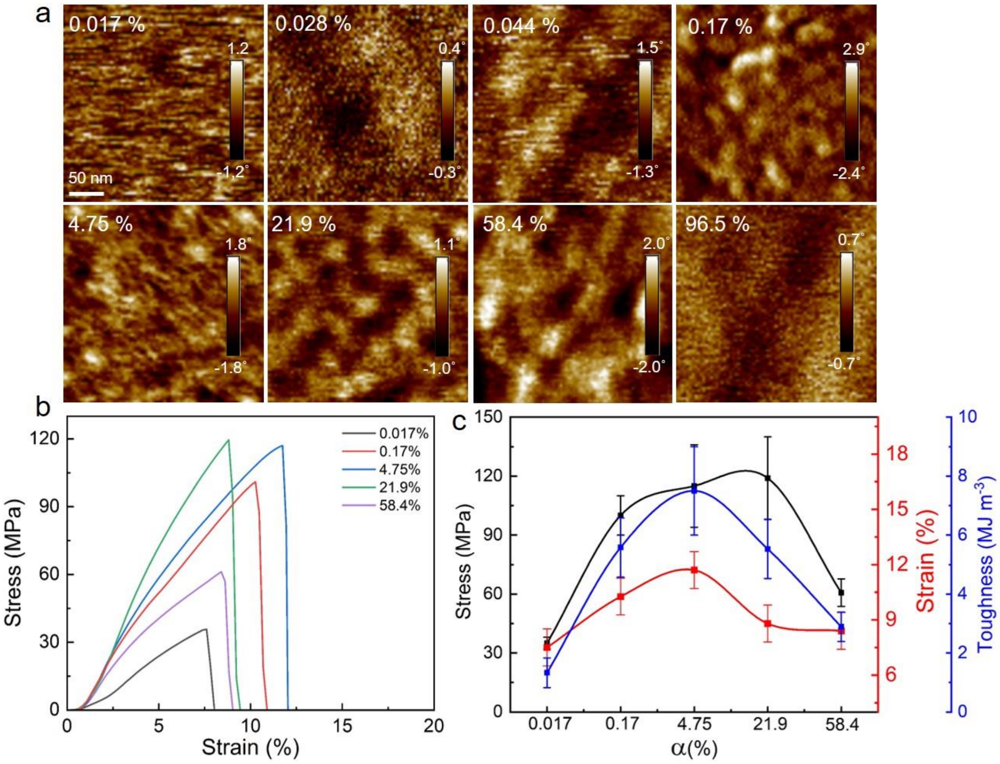

Supplementary Figure 15. AFM phase images and mechanical properties of the polyacrylic acid films. (**a**) AFM phase images of the surface of polyacrylic acid films (20 mm × 20 mm × 12 μm) with different α values that were prepared by bi-axial stretching of a polyacrylic gel (10 mm × 10 mm × 0.2 mm); (**b**) The stress–strain curves and (**c**) breaking stress, fracture strain, and toughness of polyacrylic acid films with different α. The strain rate is 50 mm min^{-1}. The error bars for (c) represent mean ± SD (n=5 independent samples).

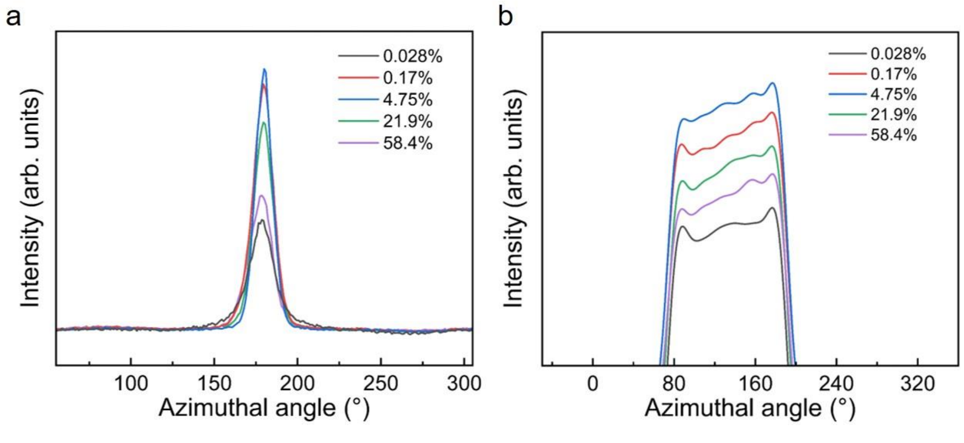

Supplementary Figure 16. Azimuthal-integrated intensity distribution curve from 2D SAXS (a) and 2D WAXS (b) of PAF_{α} with different α values.

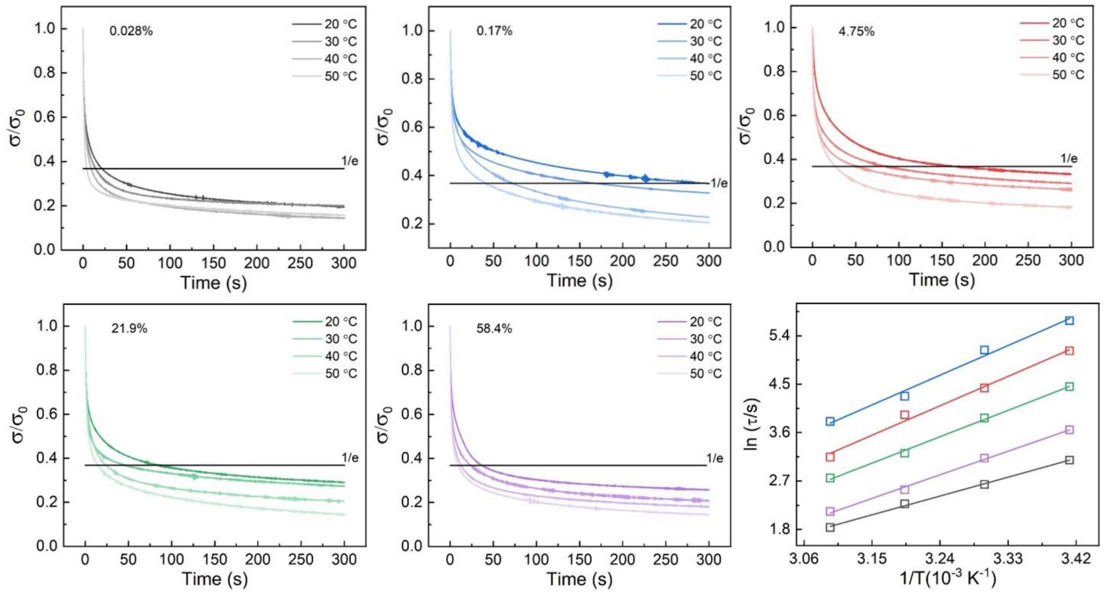

Supplementary Figure 17. Stress relaxation curves of the PAF$_{\alpha}$ fixed on the mechanical tester with 5% pre-strain at different temperatures, and the fitted results according to the *Arrhenius* equation for obtaining the *E*$_{r}$.

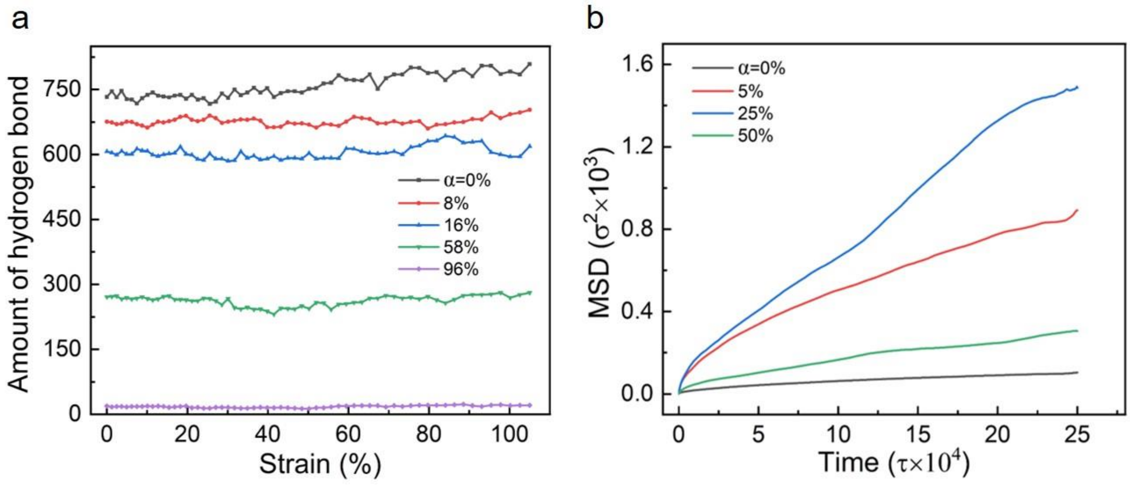

Supplementary Figure 18. (a) Amount of hydrogen bond and (b) Mean squared displacement (MSD) of the polymer centers of mass for polyacrylic systems at various dissociation degrees.

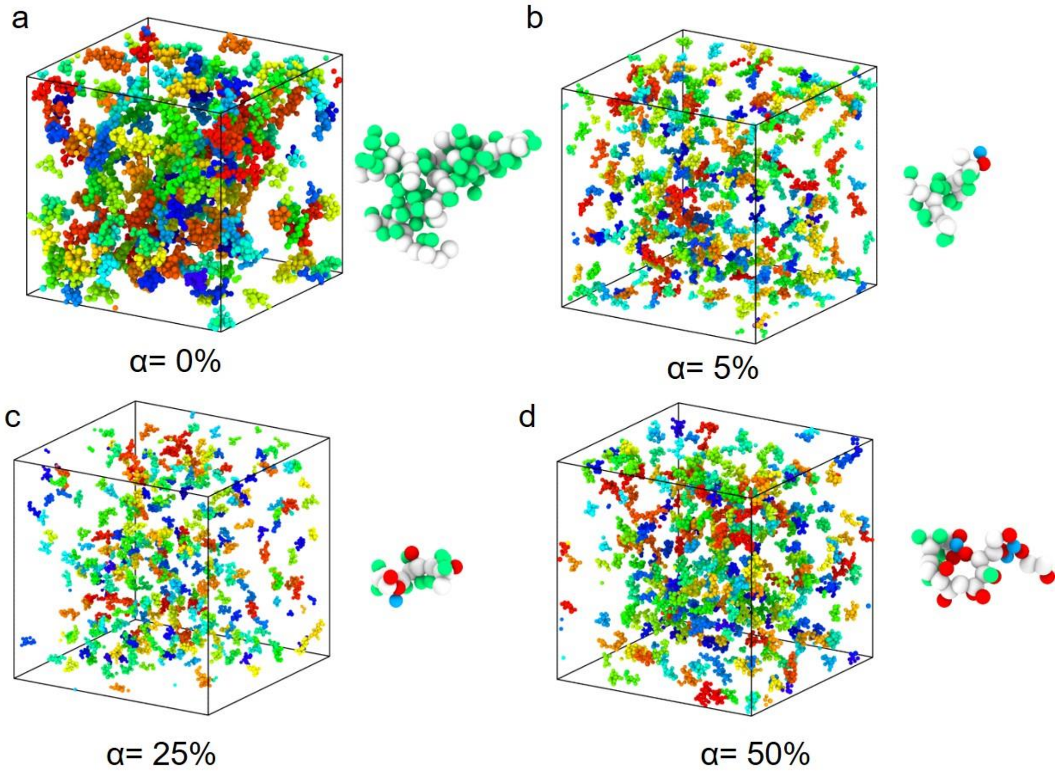

Supplementary Figure 19. Snapshots of all clusters of PAF_{α} for different α values in coarse-grained MD simulations. The largest 300 clusters were displayed and assigned with unique colors, allowing different clusters to be easily distinguished.

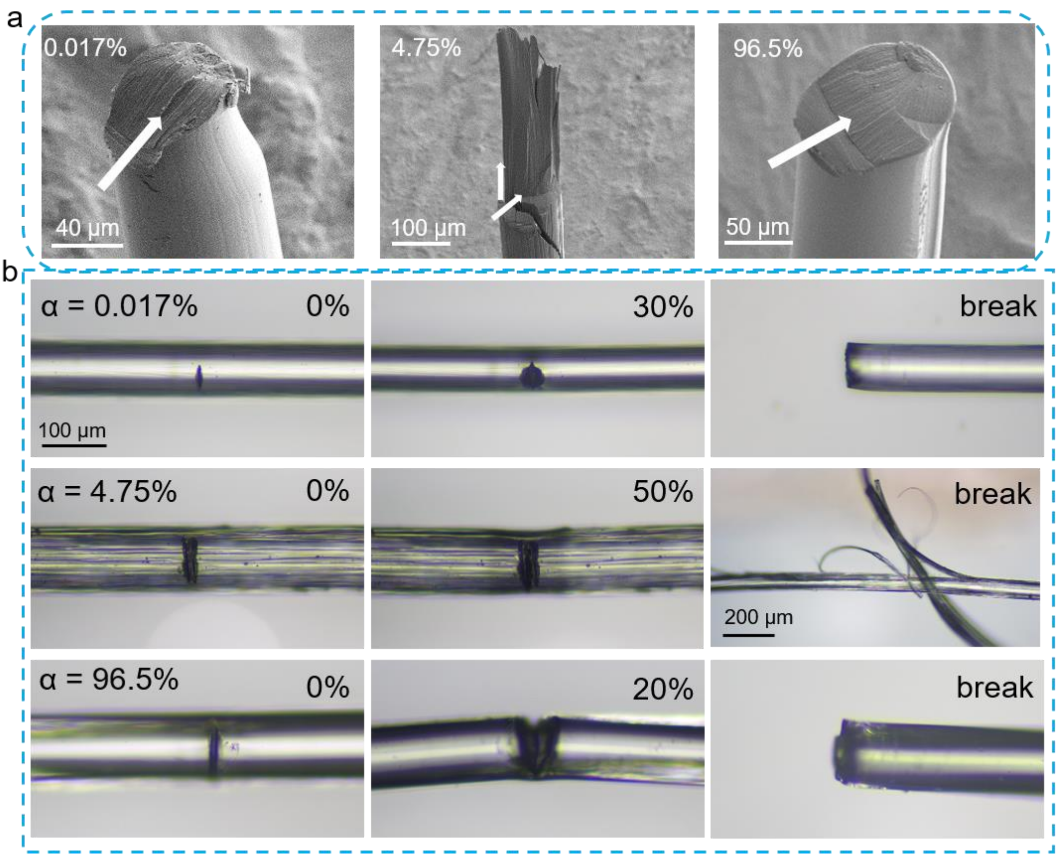

Supplementary Figure 20. (a) SEM images of the notched PAF$_{\alpha}$ after breakage and (b) the fracture process of the notched PAF$_{\alpha}$ during stretching.

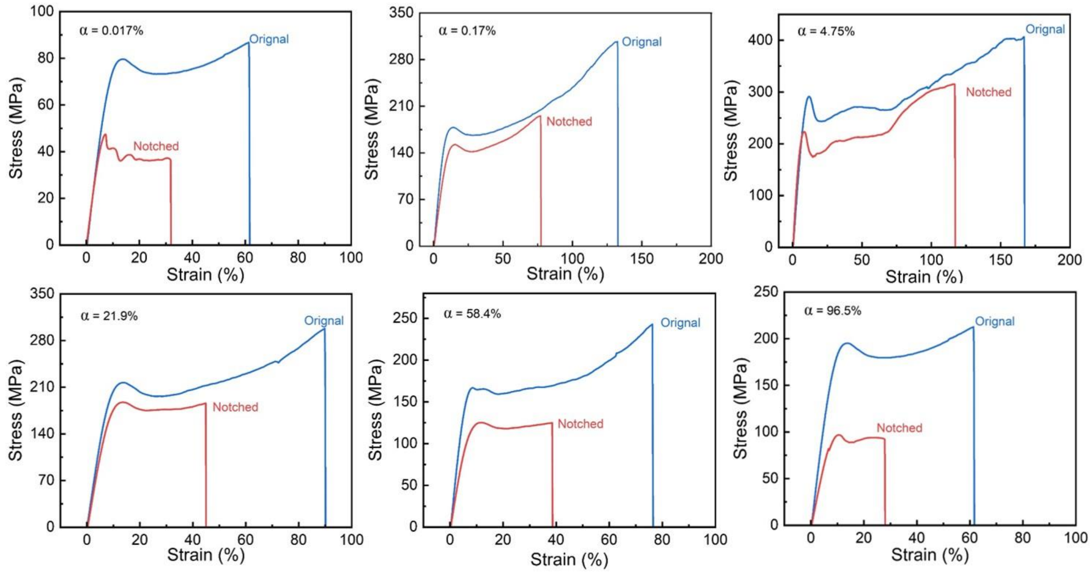

Supplementary Figure 21. Stress–strain curves of the original and notched PAF$_{\alpha}$ for different α values. The notch is 15 μm and the fiber diameter is 100 μm. The stretch rate is 5 mm min^{-1}.

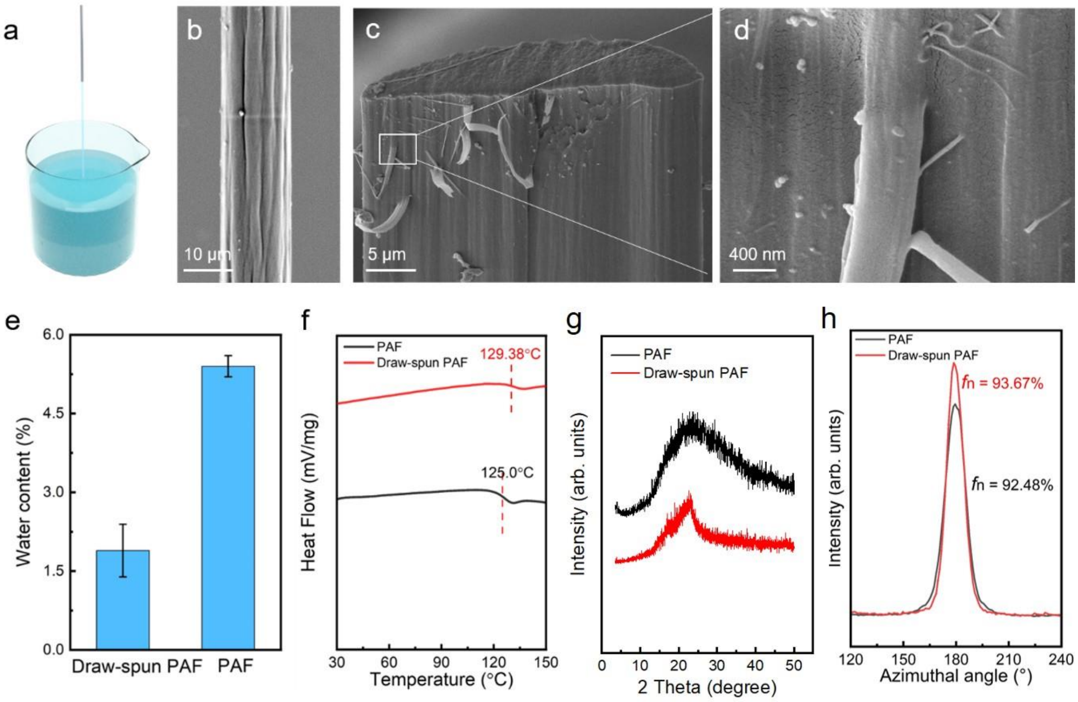

Supplementary Figure 22. The preparation and characterization of drawing-spinning of PAF$_{\alpha}$. (**a**) The illustration of drawing-spinning of a PAF$_{\alpha}$; (**b–d**) SEM images of the surface view, low and high magnified longitudinal sectional view of PAF$_{0.17\%}$; (**e**) water content, (**f**) DSC, (**g**) XRD and (**h**) Azimuthal-integrated intensity distribution curves obtained from the 2D SAXS of PAF$_{0.17\%}$ prepared by draw-spinning in comparison with that prepared in a capillary tube.

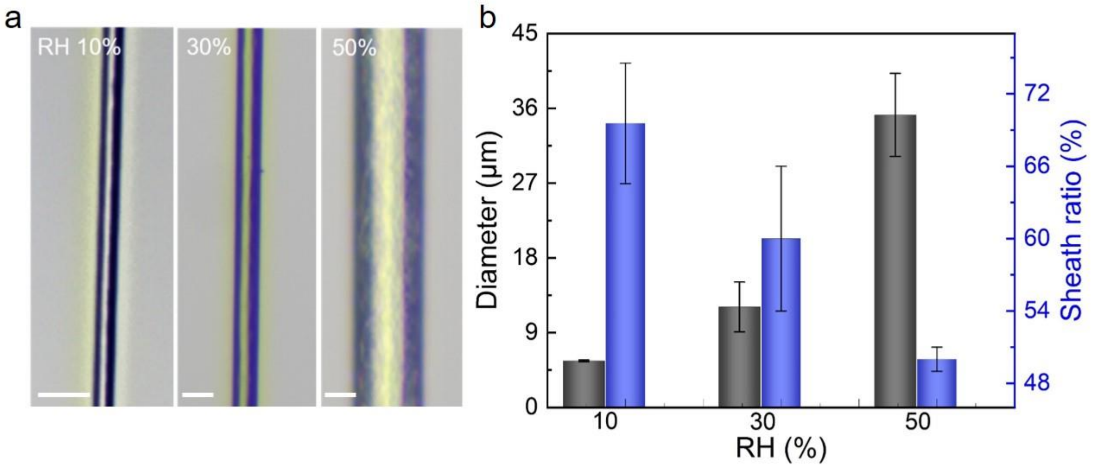

Supplementary Figure 23. (a) Metallographic microscopy images of the drawn-spun PAF$_{0.17\%}$ fiber at the different RH. Scale bar: 15 µm. (b) The fiber diameter and sheath ratio of the drawn-spun PAF$_{0.17\%}$ fiber at the different RH. The error bars for (b) represent mean ± SD (*n*=3 independent samples).

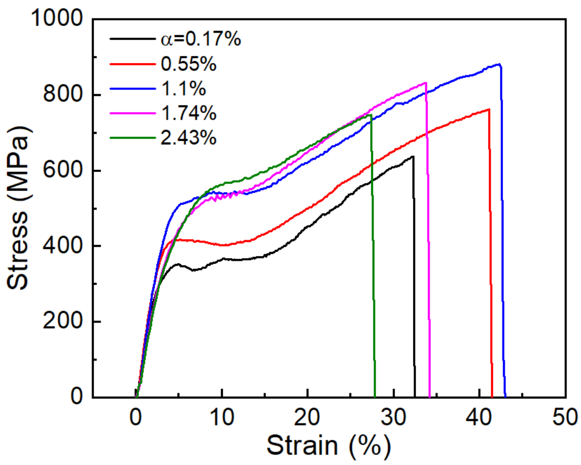

Supplementary Figure 24. Stress–strain curves of the draw-spun PAF_α for different α values. The fiber diameter was 5 μm. The stretch rate in the mechanical tests was 20 mm min^{-1}.

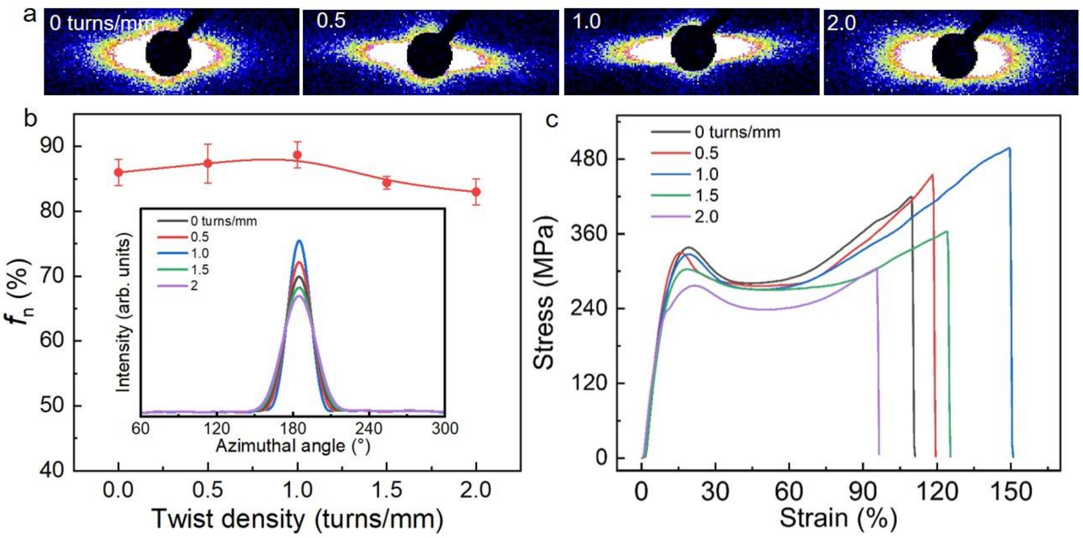

Supplementary Figure 25. (a) 2D SAXS patterns, (b) alignment degree of nanofibrils, (c) stress–strain curves of $PAF_{4.75\%}$ for different twist densities. The inset in (b) shows the azimuthal-integrated intensity distribution curves obtained from 2D SAXS patterns of $PAF_{4.75\%}$ with different twist densities. The stretch rate in the mechanical tests was 100 mm min^{-1}. The error bars for (b) represent mean ± SD (n=3 independent samples).

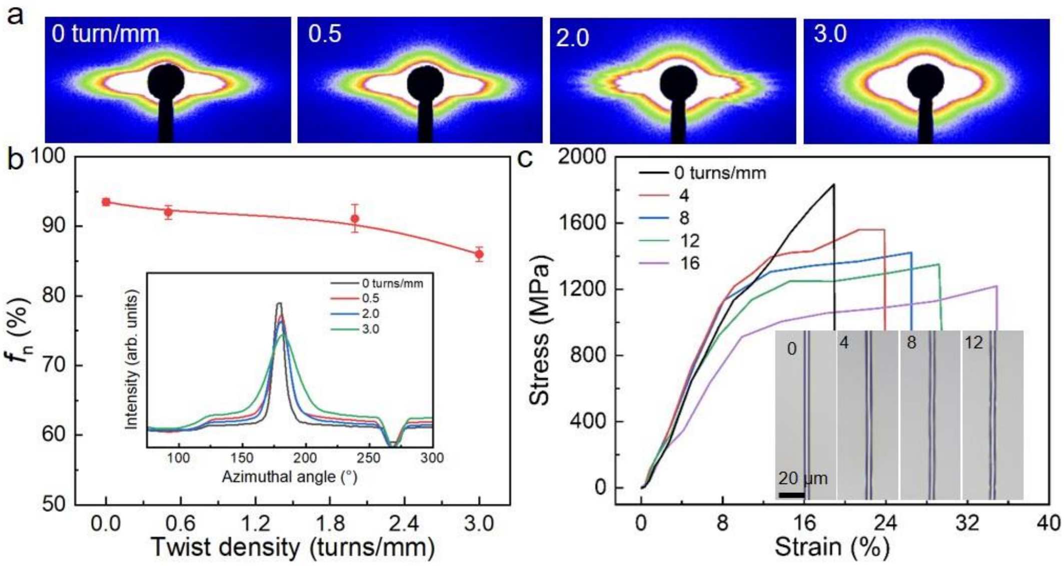

Supplementary Figure 26. (a) 2D SAXS patterns, (b) alignment degree of nanofibrils, (c) stress–strain curves of drawn-spun $PAF_{1.1\%}$ for different twist densities. The inset in (b) shows the azimuthal-integrated intensity distribution curves obtained from 2D SAXS patterns and the inset in (c) shows metallographic microscopy images of drawn-spun $PAF_{1.1\%}$ with different twist densities. The fiber diameter for (a) was 30 μm. The fiber diameter for (c) was 5 μm. The stretch rate in the mechanical tests was 500 mm min^{-1}. The error bars for (b) represent mean ± SD (n=3 independent samples).

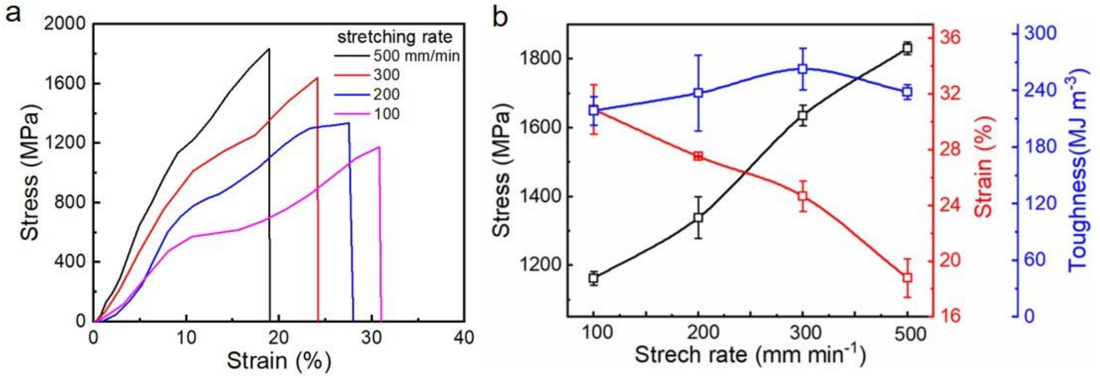

Supplementary Figure 27. (a) Stress–strain curves and (b) breaking stress, breaking strain, and toughness of the draw-spun $PAF_{1.1\%}$ for stretching rate. The fiber diameter was 5 μm. The error bars for (b) represent mean ± SD (n=5 independent

samples).

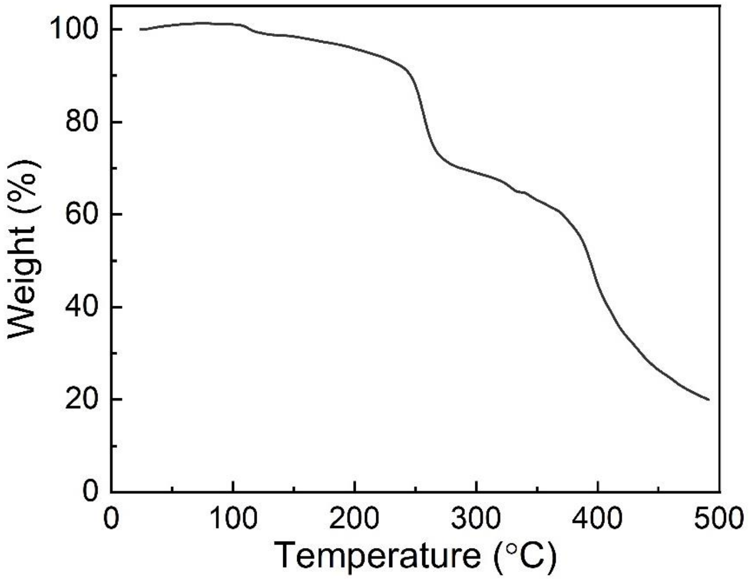

Supplementary Figure 28. The TGA of the $PAF_{4.75\%}$ at a temperature ramping rate of 10°C min^{-1} in N_2 atmosphere.

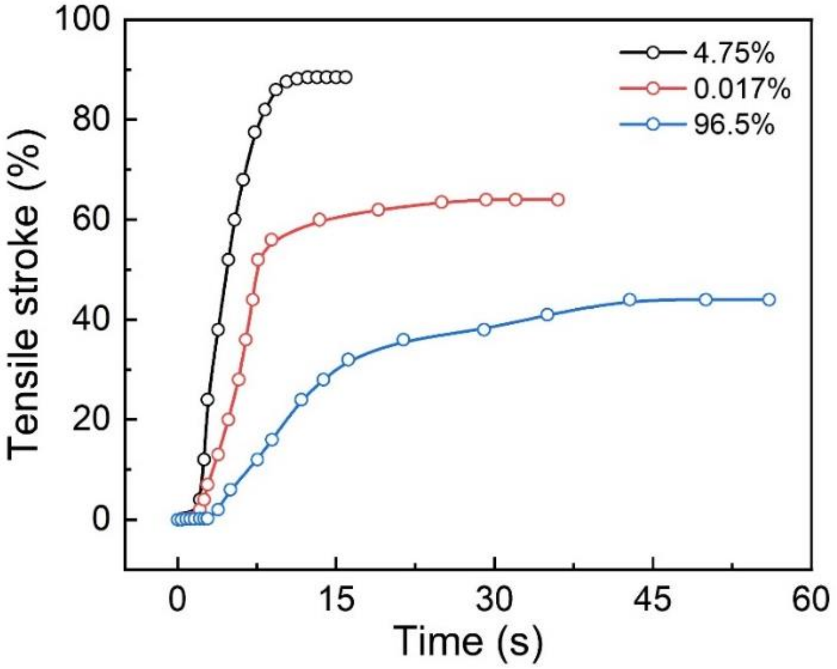

Supplementary Figure 29. The actuation stroke of a 95-μm-diameter load-free PAF_{α} with different α values for the actuation temperature of 100°C.

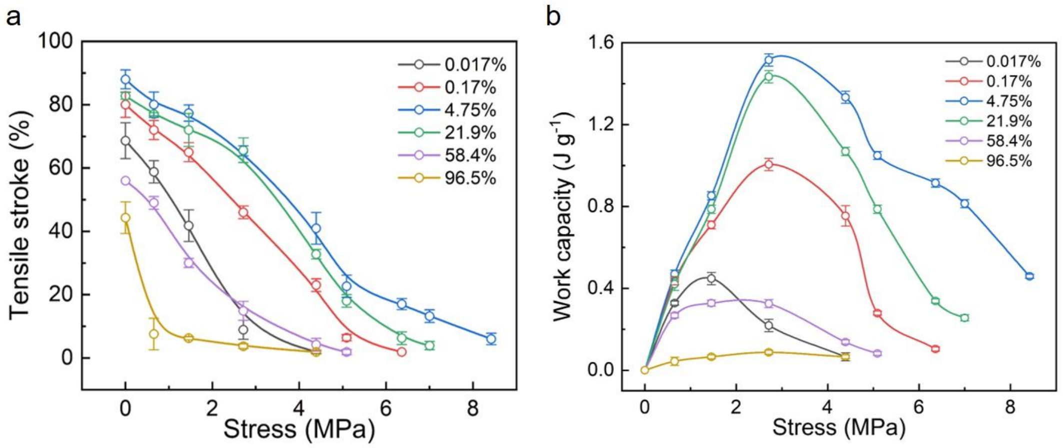

Supplementary Figure 30. (a) Actuation stroke and (b) work capacity of the PAF_{α} with different α values as a function of isobarically loaded stress. The actuation temperature was 100°C. The error bars represent mean ± SD (n=3 independent samples).

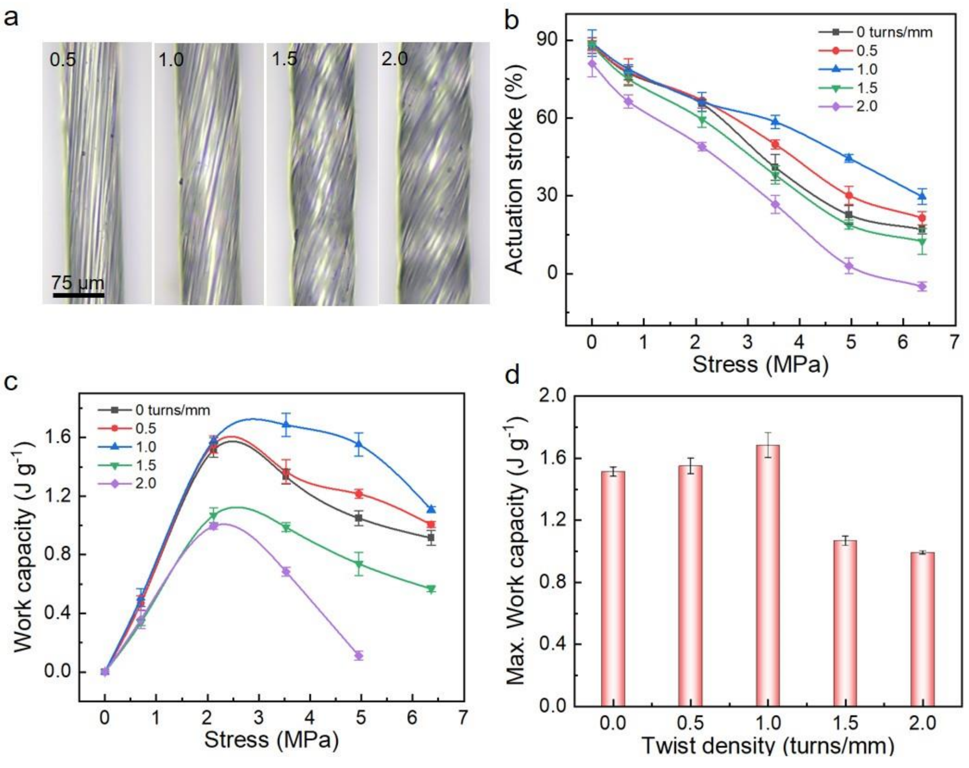

Supplementary Figure 31. The actuation properties of the $PAF_{4.75\%}$ with different twist densities. (**a**) Metallographic microscopy images of the $PAF_{4.75\%}$ fiber with different twist densities. (**b**) Actuation stroke and (**c**) work capacity of the $PAF_{4.75\%}$ as a function of isobarically loaded stress for different twist densities. (**d**) The maximum work capacity of the $PAF_{4.75\%}$ as a function of twist densities. The actuation temperature was 100°C. The error bars for (b), (c) and (d) represent mean ± SD (n=3 independent

samples).

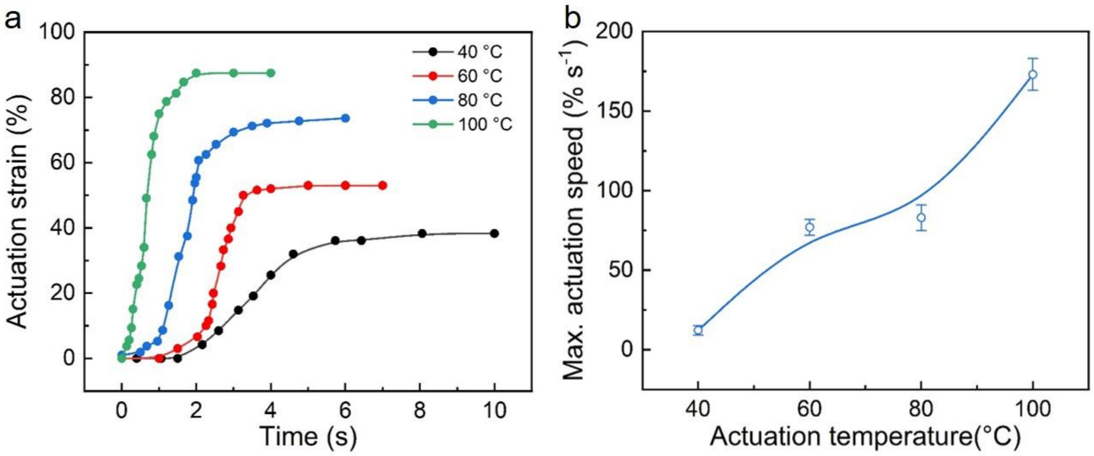

Supplementary Figure 32. (a) The actuation strain of drawn-spun $PAF_{1.1\%}$ as a function time at different actuation temperatures. (b) The maximum actuation speed of drawn-spun $PAF_{1.1\%}$ as a function of actuation temperature. The diameter of $PAF_{1.1\%}$ is 30 μm. The error bars for (b) represent mean ± SD (*n*=3 independent samples).

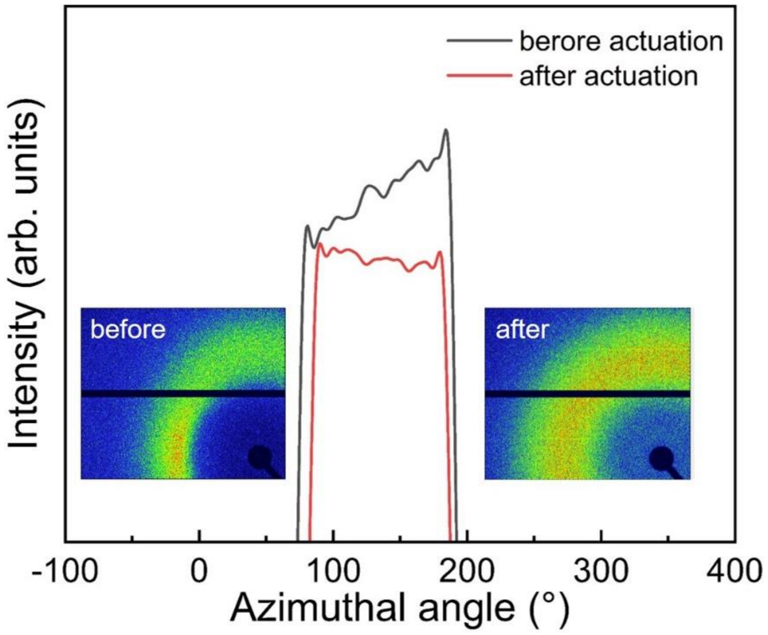

Supplementary Figure 33. Azimuthal-integrated intensity distribution curve from 2D WAXS of $PAF_{4.745\%}$ before and after actuation.

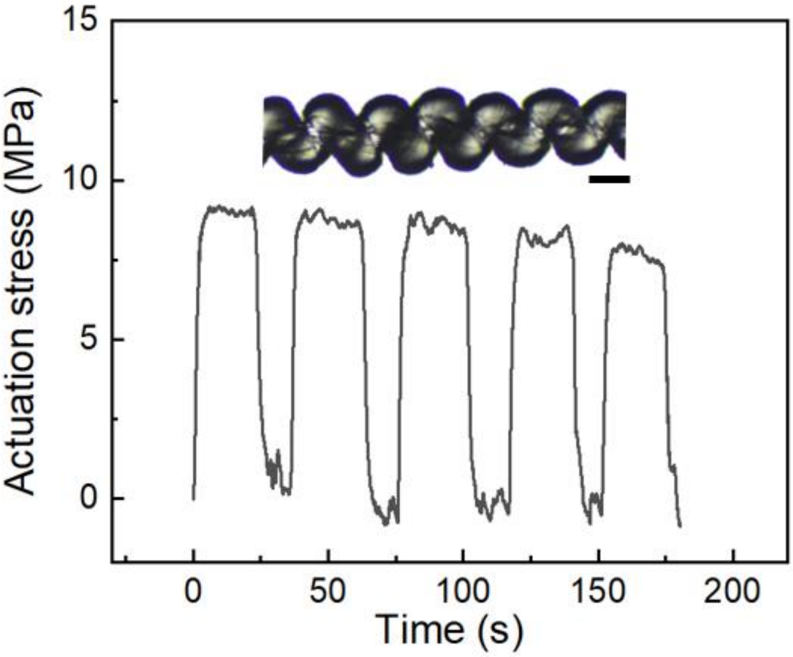

Supplementary Figure 34. Representative curve of the contractile stress of self-coiled $PAF_{4.75\%}$ artificial muscle in response to rapid increase in temperature from 25°C to 120°C via two-parallel heating plates. The twist density was 4.5 turns mm^{-1}, the fiber diameter was 95-μm, the spring index was 1.17, and the inserted twist was preserved by cross-linking using Zr^{4+} to obtain a self-supporting artificial muscle. Scale bar: 100 μm.

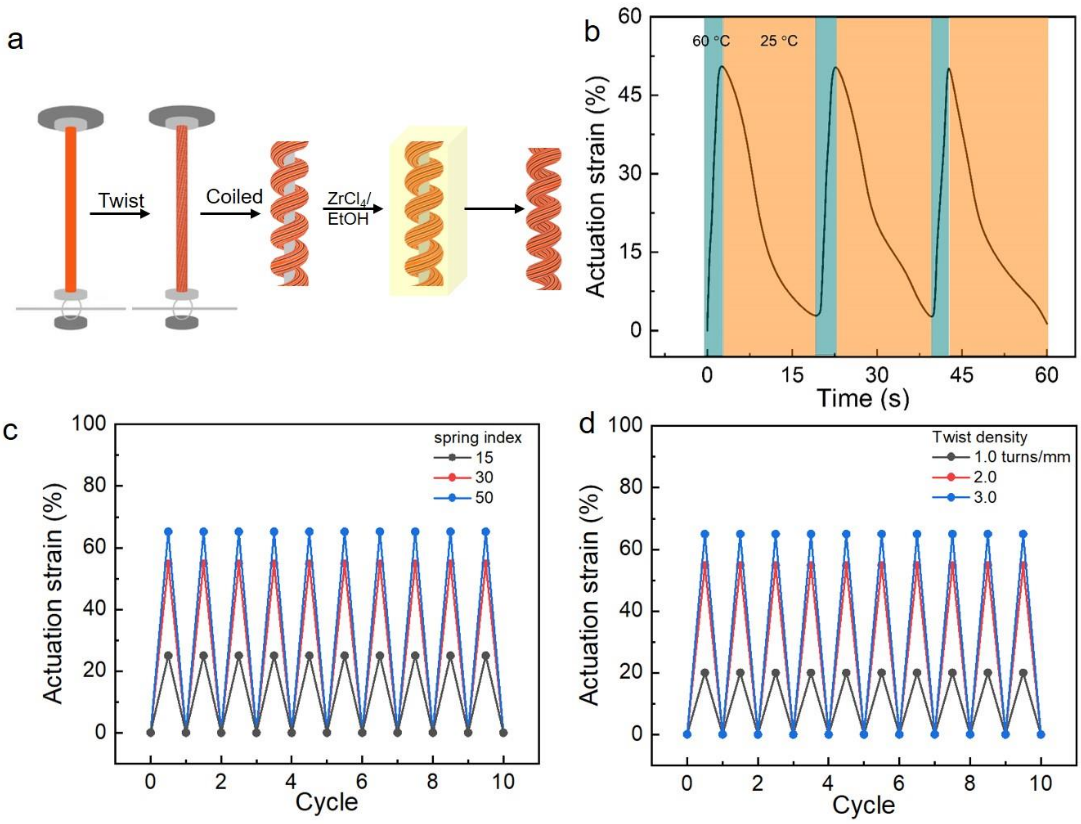

Supplementary Figure 35. The fabrication and actuation properties of the single homochiral hydrogel fiber artificial muscles. (**a**) Schematic illustration of the fabrication of the single homochiral hydrogel fiber artificial muscles. (**b**) Three cycle of the actuation stroke as a function of time for the homochiral hydrogel fiber coils in response to the temperature changes between 60°C and 25°C. The twist density is 2.0 turns mm^{-1} and the spring index is 30. (**c**) Actuator performance for the coiled homochiral hydrogel fiber muscles as a function of (c) spring index and (d) twist density.

3. Supplementary Tables

Supplementary Table 1. Possible choices of the Martini 3 beads for each chemical group, molecular weight (ignoring the ends), and approximate reduced mass (as used in the simulations).

Atomic Structure	Bead Type	Reduced Mass	Atomic Mass [g mol^{-1}]
$(CH2-CH2)_2$	R-C2	1.25	56
–COOH	S-P2	1.0	45
$-COO^-$	S-Qn (−1)	1.0	44
K+	S-Qp (+1)	1.0	40

Supplementary Table 2. LJ zero-crossing distance (σ_{ij}) and well depth (ϵ_{ij}) parameter matrix for the chosen bead types (the reduced distance σ_{ij} is 4.0 Å, and the reduced energy ϵ is1.97 kJ mol^{-1}).

Atomic Structure	CH_2–CH_2–CH_2–CH_2	–COOH	$-COO^-$	Na^+/K^+
$(CH2-CH2)_2$	C2–C2<br>4.7 Å 3.50 kJ mol^{-1}	C2−SP2<br>4.4 Å 1.97 kJ mol^{-1}	C2−SQn<br>5.15 Å<br>2.19 kJ mol^{-1}	C2−SQp<br>5.15 Å<br>2.19 kJ mol^{-1}
–COOH		SP2−SP2<br>4.0 Å<br>3.25 kJ mol^{-1}	SP2−SQn<br>4.0 Å<br>5.49 kJ mol^{-1}	SP2−SQp<br>4.0 Å<br>5.49 kJ mol^{-1}
$-COO^-$			SQn−SQn<br>4.0 Å<br>2.30 kJ mol^{-1}	SQn−SQp<br>4.0 Å<br>3.15 kJ mol^{-1}
Na^+/K^+				SQp−SQp<br>4.0 Å<br>2.30 kJ mol^{-1}

Supplementary Table 3. The molar ratio, pH, and α values for $PAF_α$ with different feed ratios.

	n_{AA}: n_{HCl}				n_{AA}: n_{NaOH}					
Molar ratio	10:1	20:1	40:1	1:0	20:1	10:1	8:1	4:1	2:1	1:1
pH	0.8	1.1	1.3	2	2.9	3.45	3.7	4.2	4.9	6.2
α (%)	0.017	0.028	0.044	0.17	1.38	4.75	8.14	21.9	58.4	96.5

Note: n_{AA}, n_{HCl}, and n_{NaOH} denote the molar amount of the AA, HCl, and NaOH during polymerizaiton, respectively, in this and the following tables.

Supplementary Table 4. The water content of the 80-μm-diameter $PAF_α$ for different α values dried at 60°C for 3 h.

α (%)	0.017	0.028	0.17	4.75	21.9	58.4	96.5
Water content (%)	5.8	5.6	5.4	5.6	6.6	8.8	13.2

Supplementary Table 5. The molar ratio and α values for $PAF_α$ prepared by adding different bases.

	n_{AA}: n_{LiOH}	n_{AA}: n_{NaOH}	n_{AA}: n_{KOH}	n_{AA}: $n_{NH3·H2O}$
Molar ratio	9:1	9:1	9:1	9:1
α (%)	4.75	5.3	5.9	2.95

Note: n_{LiOH}, n_{KOH}, and $n_{NH3·H2O}$ denote the molar amount of the LiOH, KOH, and NH_3•H_2O during polymerization, respectively.

Supplementary Table 6. The molar ratio and α values for $PAF_α$ prepared by adding different acids.

	n_{AA}: $n_{B(OH)3}$	n_{AA}: $n_{CH3COOH}$	n_{AA}: $n_{CH3SO3H}$	n_{AA}: n_{HCl}
Molar ratio	8:1	8:1	8:1	8:1
α (%)	0.17	0.14	0.07	0.01

Note: $n_{B(OH)3}$, $n_{CH3COOH}$, $n_{CH3SO3H}$, n_{HCl}, denote the molar amount of the $B(OH)_3$, CH_3COOH, CH_3SO_3H, during polymerization, respectively.

Supplementary Table 7. Comparison of the mechanical properties of typical artificial spider silks reported in the literatures.

Artificial spider silk	Stress (MPa)	Strain (%)	Toughness (MJ m^{-3})	Ref.
	603	30	113	6
	279	30	51.8	7
Protein fiber	167	197	249	8
	866	20	160	9
	255	45	90	10
	478	9	31.8	11
Composite fiber	535	12.2	38.3	12
	949	80	296	13
	1130	35.4	305.4	14
	100	750	387	15
	442	130	640	16
PU fiber	75.6	1520	390.2	17
	60.7	1200	263.6	18
	52.4	2100	363.8	19
Hydrogel fiber	193	18.1	22.8	20
	895	44.2	214	21
**This work**	**530-1831**	**176-18.8**	**700-238**	

Supplementary Table 8. Comparison of the fracture energy of the typical tough materials reported in literatures.

Tough materials	Stress (MPa)	Strain (%)	fracture energy (MJ m^{-2})	Ref.
IPDI-SPU elastomer	17.5	1398	0.215	22
PVA/HCPE elastomer	100	–	0.13	23
Octuple HB elastomer	1.5	100	0.017	24
Microsphere-reinforced ionogel	0.3	3000	0.087	25
P(AAm-co-AA) ionogel	12.6	600	0.024	26
PAAm/alginate hydrogel	0.175	1600	0.0087	27
Polyampholyte hydrogel	2	700	0.004	28
hydrogel microfiber	10.5	219	0.187	29
**This work**	**320**	**115**	**1.65**	

Supplementary Table 9. Comparison of the mechanical properties and the nanofibril size of typical fibers with nanofibrils in literature.

Artificial spider silk	Nanofibrils sizes(nm)	Stress (GPa)	Strain (%)	Toughness (MJ m^{-3})	Ref.
Supermolecular fiber	100	0.193	18.1	22.8	20
Regenerated B. mori silk fiber	100	0.4	15	–	30
Silk fibroin fiber	65	0.008	4	–	31
PVA/Alg/HAP composite fiber	1000	0.949	80	296	13
Regenerated spidroins fiber	100	0.3	48	100	32
Protein fibers	20	0.016	4	0.7	33
Protein/genipin fibers	38.4	0.0204	2	0.3	34
Protein/alginate fibers	1500	0.058	7	–	35
Alginate hydrogel ionotronic fiber	250	0.0006	100	–	36
**This work**	**12.5**	**1.83**	**18.8**	**238**	

Supplementary Table 10. Comparison of the work capacity and actuation stroke of typical artificial muscles in literature.

**artificial muscles**	**Break stress (MPa)**	**Actuation stress (MPa)**	**Actuation stroke (%)**	**Work capacity ($J\ g^{-1}$)**	**Ref.**
PAA-Polyester fiber	-	1.0	70	1.0	37
PAA/Ca(CH3COO)2 fiber	14	-	52.7	0.0452	38
PAA/cotton fiber	150	0.03	61	0.0061	39
PAA/CNT fiber	200	0.01	56	0.0091	40
PAAM/PAA/TCNC fiber	65	0.24	36	0.0424	41
PAM/PAA/PSMA/Q-TCNC fiber	32.6	3.0	75	0.21	42
Nylon/BA-PA fiber	0.73	0.08	1.4	0.13	43
Linen yarn	-	-	10	0.11	44
Human hair	200	0.25	20	0.006	46
**This work**	**530-1800**	**6.22-65**	**11-56.3**	**1.58-2.77**	

Supplementary Table 11. Comparison of the actuation performance of typical thermally supercontraction artificial muscles in literature.

Materials	Break stress (MPa)	Actuation stroke (%)	Actuation stress (MPa)	Work capacity (J g^{-1})	Ref.
Mammalian skeletal muscles	0.47	40	0.35	0.039	47
Semicrystallie polymer fibers	-	49	1.0	2.48	48
COCe-PE bimorph fiber	25.8	47.7	5.0	7.42	49
Block polymer	146.2	30.8	5.5	0.506	47
Supramolecular Nanostructures ploymer	70	300	13.1	-	50
LCE-Graphene	90	45	1.23	0.65	51
LCE	80	274	1.3	0.73	52
	2.53	40	0.35	1.83	53
	3.2	296	0.1	0.296	54
PU	25	24	1.0	0.708	55
	12	65	0.002	0.717	56
	12	-	0.023	0.0725	57
PU/LCE/AU	12	66	0.001	0.87	58
**This work**	**530-1800**	**11-56.3**	**6.22-65**	**1.58-2.77**	

4. Supplementary Data

Supplementary Data 1. The initial and final configurations of the cluster of different α values via coarse-grained molecular simulations.

Supplementary References

1. Souza, P. C. *et al.* Martini 3: a general purpose force field for coarse-grained molecular dynamics. *Nat Methods* **18**, 382–388 (2021).
2. Liesen, N. T. *et al.* The influence of spacer composition on thermomechanical properties, crystallinity, and morphology in ionene segmented copolymers. *Soft Matter* **17**, 5508–5523 (2021).
3. Sampath, J., & Hall, L.M. Effect of neutralization on the structure and dynamics of model ionomer melts. *Macromolecules* **51**, 626–637 (2018).
4. Plimpton, S. Fast parallel algorithms for short-range molecular dynamics. *J. Comp. Phys.* **117,** 1–19 (1995).
5. Plimpton, S., Pollock, R., & Stevens, M. Particle-mesh ewald and rRESPA for parallel molecular dynamics simulations. *PPSC* (1997).
6. Li, Y. *et al.* Bioinspired and mechanically strong fibers based on engineered non-spider chimeric proteins. *Angew. Chem. Int. Ed.* **132**, 1–6 (2020).
7. He, H. *et al.* Mechanically strong globular-protein-based fibers obtained using a microfluidic spinning technique. *Angew. Chem. Int. Ed.* **59**, 4344–4348 (2020).
8. Zhu, H. *et al.* Tough synthetic spider-silk fibers obtained by titanium dioxide incorporation and formaldehyde cross-linking in a simple wet-spinning process. *Biochimie* **175**, 77–84 (2020).
9. Li, J. *et al.* Microbially synthesized polymeric amyloid fiber promotes β-nanocrystal formation and displays gigapascal tensile strength. *ACS Nano* **15**, 11843–11853 (2021).
10. Yazawa, K. *et al.* Combination of amorphous silk fiber spinning and postspinning crystallization for tough regenerated silk fibers. *Biomacromolecules* **19**, 2227–2237 (2018).
11. Mohammadi, P. *et al.* Biomimetic composites with enhanced toughening using silk-inspired triblock proteins and aligned nanocellulose reinforcements. *Sci. Adv.* **5**, eaaw2541 (2019).
12. Gao, H.-L. *et al.* Bioinspired hierarchical helical nanocomposite macrofibers based on bacterial cellulose nanofibers. *Natl. Sci. Rev.* **7**, 73–83 (2020).
13. Yu, Y. *et al.* Biomimetic mineralized organic–inorganic hybrid macrofiber with spider silk-like supertoughness. *Adv. Funct. Mater.* **30**, 1908556 (2020).
14. Cao, C. *et al.* Strong reduced graphene oxide coated bombyx mori silk. *Adv. Funct. Mater.* **31**, 2102923 (2021).
15. Gu, L., Jiang, Y., & Hu, J. Scalable spider-silk-like supertough fibers using a pseudoprotein polymer. *Adv. Mater.* **31**, 1904311 (2019).
16. Xi, P. *et al.* Strategy to fabricate a strong and supertough bio-inspired fiber with organic–inorganic networks in a green and scalable way. *ACS Nano* **15**, 16478–16487 (2021).
17. Li, Z. *et al.* Healable and recyclable elastomers with record-high mechanical

robustness, unprecedented crack tolerance, and superhigh elastic restorability. *Adv. Mater.* **33**, 2101498 (2021).

18. Liu, W., Fang, C., Wang, S., Huang, J. & Qiu, X. High-performance lignin-containing polyurethane elastomers with dynamic covalent polymer networks. *Macromolecules* **52**, 6474–6484 (2019).
19. Wang, X. *et al.* Healable, recyclable, and mechanically tough polyurethane elastomers with exceptional damage tolerance. *Adv. Mater.* **32**, 2005759 (2020).
20. Wu, Y. *et al.* Bioinspired supramolecular fibers drawn from a multiphase self-assembled hydrogel. *PANS* **114**, 8163–8168 (2017).
21. Dou, Y. *et al.* Artificial spider silk from ion-doped and twisted core-sheath hydrogel fibres. *Nat. Commun.* **10**, 5293 (2019).
22. Li, Z. *et al.* Healable and recyclable elastomers with record-high mechanical robustness, unprecedented crack tolerance, and superhigh elastic restorability. *Adv. Mater.* **33**, 2101498 (2021).
23. Liu, L. *et al.* Dynamic nanoconfinement enabled highly stretchable and supratough polymeric materials with desirable healability and biocompatibility. *Adv. Mater.* **33**, 2105829 (2021).
24. Zhuo, Y. *et al.* Simultaneously toughening and stiffening elastomers with octuple hydrogen bonding. *Adv. Mater.* **33**, 2008523 (2021).
25. Li, W. *et al.* Recyclable, healable, and tough ionogels insensitive to crack propagation. *Adv. Mater.* **34**, 2203049 (2022).
26. Wang, M. *et al.* Tough and stretchable ionogels by in situ phase separation. *Nat. Mater.* **21**, 359–365 (2022).
27. Sun, J.-Y. *et al.* Highly stretchable and tough hydrogels. *Nature* **489**, 133–136 (2012).
28. Sun, T. L. *et al.* Physical hydrogels composed of polyampholytes demonstrate high toughness and viscoelasticity. *Nat. Mater.* **12**, 932–937 (2013).
29. Shi, Y., Wu, B., Sun, S., & Wu, P. Aqueous spinning of robust, self-healable, and crack-resistant hydrogel microfibers enabled by hydrogen bond nanoconfinement. *Nat. Commun.* **14**, 1370 (2023).
30. Wan, Q. *et al.* Mesoscale structure development reveals when a silkworm silk is spun. *Nat. Commun.* **12**, 3711 (2021).
31. Fan, L., Li, J-L, Cai, Z., & Wang, X. Bioactive hierarchical silk fibers created by bioinspired self-assembly. *Nat. Commun.* **12**, 2375 (2021).
32. Hu, C.-F., *et al.* Unconventional spidroin assemblies in aqueous dope for spinning into tough synthetic fibers. *ACS Biomater. Sci. Eng.* **7**, 3608–3617 (2021).
33. Mohammadi, P. *et al.* Phase transitions as intermediate steps in the formation of molecularly engineered protein fibers. *Commun Biol* **1**, 86 (2018).
34. Ye, X. *et al.* Robust assembly of cross-linked protein nanofibrils into hierarchically structured microfibers. *ACS Nano* **16**, 12471–12479 (2022).
35. Kamada, A. *et al.* Modulating the Mechanical Performance of Macroscale Fibers through Shear-Induced Alignment and Assembly of Protein Nanofibrils. *Small* **16**,

1904190 (2020).

36. Chen, Z. *et al.* Mechanically and electrically biocompatible hydrogel ionotronic fibers for fabricating structurally stable implants and enabling noncontact physioelectrical modulation. *Mater. Horiz.* **9**, 1735−1749 (2022).

37. Spinks, G. M., Martino, N. D., Naficy, S., Shepherd, D. J. & Foroughi, J. Dual high-stroke and high−work capacity artificial muscles inspired by DNA supercoiling. *Sci. Robot.* **6**, eabf4788 (2021).

38. Hua, L., Zhao, C., Guan, X., Lu, J. & Zhang, J. Cold-induced shape memory hydrogels for strong and programmable artificial muscles. *Sci. China Mater.* **65**, 2274−2280 (2022).

39. Khan, A. Q. *et al.* Spider silk supercontraction-inspired cotton-hydrogel self-adapting textiles. *Adv. Fiber Mater.* **4**, 1572−1583 (2022).

40. You, C. *et al.* Highly improved water tolerance of hydrogel fibers with a carbon nanotube sheath for rotational, contractile and elongational actuation. *J. Mater. Chem. A.* **9**, 10240−10250 (2021).

41. Cui, Y., Li, D., Gong, C., & Chang, C. Bioinspired shape memory hydrogel artificial muscles driven by solvents. *ACS Nano* **15**, 13712−13720 (2021).

42. Chen, M., Cui, Y., Wang, Y., & Chang, C. Triple physically cross-linked hydrogel artificial muscles with high-stroke and high-work capacity. *Chem. Eng. J.* **453**, 139893 (2023).

43. Sim, H. J. *et al.* Self-helical fiber for glucose-responsive artificial muscle. *ACS Appl. Mater. Interfaces.* **12**, 20228−20233 (2020).

44. Wang, Y., & Miao, M. Helical shape linen artificial muscles responsive to water. *Smart Mater. Struct.* **30**, 075031 (2021).

45. Wang, Y. *et al.* Humidity-and water-responsive torsional and contractile lotus fiber yarn artificial muscles. *ACS Appl. Mater. Interfaces.* **13**, 6642−6649 (2021).

46. Leng, X. *et al.* Tuning the reversibility of hair artificial muscles by disulfide crcoss-linking for sensors, switches, and soft robotics. *Mater. Horiz.* **8**, 1538−1546 (2021).

47. Lang, C. *et al.* Nanostructured block copolymer muscles. *Nat. Nanotechnol.* **17**, 752 (2022).

48. Haines, C. S. *et al.* Artificial Muscles from Fishing Line and Sewing Thread. *Science* **343**, 868–872 (2014).

49. Kanik, M. *et al.* Strain-programmable fiber-based artificial muscle. *Science* **365**, 145 (2019).

50. Cooper, C. B. *et al.* High Energy Density Shape Memory Polymers Using Strain-Induced Supramolecular Nanostructures. *ACS Cent. Sci.* **7**, 1657–1667 (2021).

51. Kim, I. H. *et al.* Human-muscle-inspired single fibre actuator with reversible percolation. *Nat. Nanotechnol.* **17**, 1198–1205 (2022).

52. Kim, H. *et al.* Tough, Shape-Changing Materials: Crystallized Liquid Crystal Elastomers. *Macromolecules* **50**, 4267–4275 (2017).

53. Lu, H.-F. *et al.* Interpenetrating Liquid-Crystal Polyurethane/Polyacrylate

Elastomer with Ultrastrong Mechanical Property. *J. Am. Chem. Soc.* **141**, 14364–14369 (2019).
54. Saed, M. O. *et al.* Thiol-Acrylate Main-Chain Liquid-Crystalline Elastomers with Tunable Thermomechanical Properties and Actuation Strain. *J. Polym. Sci. Part B: Polym. Phys.* **55**, 157–168 (2017).
55. Guo, Y. *et al.* Ultra-tough and stress-free two-way shape memory polyurethane induced by polymer segment "spring". *Chem. Eng. J.* **470**, 144212 (2023).
56. Zheng, X. *et al.* High-energy-density shape memory materials with ultrahigh strain for reconfigurable artificial muscles. *J. Mater. Chem. B.* **9**, 7371–7380 (2021).
57. Rehman, H. U. *et al.* High-cycle-life and high-loading copolymer network with potential application as a soft actuator. *Materials and Design* **182**, 108010 (2019).
58. Chen, C. *et al.* Multiresponse Shape-Memory Nanocomposite with a Reversible Cycle for Powerful Artificial Muscles. *Chem. Mater.* **33**, 987–997 (2021).
