# Supplementary material for: Establishing superfine nanofibrils for robust polyelectrolyte artificial spider silk and powerful artificial muscles
Source: Nat Commun. 2024 Apr 25;15:3485. doi: 10.1038/s41467-024-47796-2 (PMC11045855; doi:10.1038/s41467-024-47796-2)
Supplement: Supplementary file 3 — Description of Additional Supplementary Files [file 41467_2024_47796_MOESM3_ESM.pdf]

Description of Additional Supplementary Files

File Name: Supplementary Data 1
Description: The initial and final configurations of the cluster of different α values via coarse-grained molecular simulations.
